# Exceptional tumor-free survival of a patient with metastatic intrahepatic cholangiocarcinoma after surgery and personalized peptide vaccination: revisiting a striking case

**DOI:** 10.1136/jitc-2025-012107

**Published:** 2025-10-09

**Authors:** Ana Maia, Juliane Schuhmacher, Silvio Nadalin, Alfred Königsrainer, Karolin Thiel, Annika Nelde, Raphael S Zinser, Christopher Schroeder, Sven Mattern, Stephan Singer, Hans Bösmüller, Hans-Georg Rammensee, Markus W Löffler, Cécile Gouttefangeas

**Affiliations:** 1 Institute of Immunology, University and University Hospital Tübingen, Tübingen, Germany; 2 Department of General, Visceral and Transplant Surgery, University Hospital Tübingen, Tübingen, Germany; 3 Cluster of Excellence iFIT (EXC2180) ‘Image-Guided and Functionally Instructed Tumor Therapies’, University of Tübingen, Tübingen, Germany; 4 Department of General, Visceral and Thoracic Surgery, Oberschwabenklinik gGmbH, St. Elisabethen-Klinikum, Ravensburg, Germany; 5 Institute of Immunology, Department of Peptide-based Immunotherapy, University and University Hospital Tübingen, Tübingen, Germany; 6 Institute of Medical Genetics and Applied Genomics, University Hospital Tübingen, Tübingen, Germany; 7 German Cancer Consortium (DKTK) and German Cancer Research Center (DKFZ), partner site Tübingen, Heidelberg, Germany; 8 Institute of Pathology and Neuropathology, University Hospital Tübingen, Tübingen, Germany; 9 Institute for Clinical and Experimental Transfusion Medicine (IKET), University Hospital Tübingen, Tübingen, Germany

**Keywords:** metastatic cholangiocarcinoma, case report, cancer vaccine, CD4^+^T cells

## Abstract

Cholangiocarcinomas are rare but aggressive liver tumors of high lethality with scarce treatment options. Here we report on the follow-up of a patient diagnosed with an intrahepatic cholangiocarcinoma who experienced repeated tumor recurrences including distant metastasis, therefore facing a dismal prognosis. At present, this patient is tumor-free for more than 8 years following repeated surgery and application of two successive personalized vaccines. In-depth functional immune cell analyses revealed a dominant CD4^+^ T-cell response against the vaccine antigens with infiltration of the tumor site and immune responses prevailing for years following the last vaccine administration. Additionally, spontaneous tumor neoantigen-specific CD4^+^ and CD8^+^ T-cell responses have been detected, which might have contributed to the outstanding outcome witnessed in this patient. This case report highlights vaccination strategies targeting non-mutated antigens as well as the increasingly recognized central role of antitumor CD4^+^ T cells.

## Introduction

 Cholangiocarcinomas (CCAs) are rare genetically and anatomically heterogeneous malignancies emerging from hepatic ductal cells with particularly poor prognosis and high mortality rates.^1^ According to their anatomical location in the biliary tree, CCAs are divided into intrahepatic (iCCAs), perihilar or distal CCAs. Meanwhile, iCCAs are recognized as a separate entity that can be subcategorized into small or large duct subtypes based on different growth patterns and distinct genetic variants. To date, surgery remains the only established curative option for CCA; however, less than one-third of the patients undergoing surgical resection with curative intent survive beyond 5 years.^2^ Therefore, metastatic and unresectable CCAs are considered a fatal incurable disease stage. Further potentially curative treatment options, including liver transplantation or immunotherapy, are uncommon and highly experimental. Tran *et al* have successfully treated a patient with metastatic CCA and substantial tumor burden with adoptive transfer of tumor-infiltrating lymphocytes containing CD4^+^ T helper 1 cells recognizing a genetic variant specifically expressed in the patient’s tumor.^3^ This and further work have triggered substantial interest in immunotherapies for CCAs.^1 4^ Nevertheless, available research suggests only limited activity of single-agent immune checkpoint blockade (ICB), except for the infrequent subtype of CCAs with mismatch repair deficiencies.^5^

We have previously reported on a patient with iCCA who has been treated with surgery and a personalized peptide vaccine as an individual treatment attempt.^6^ Back then, the patient had been without detectable disease for 3 years but eventually relapsed with another liver lesion. Surgical resection and vaccinations with an adjusted multi-peptide vaccine ensued. Meanwhile, the course of treatment extends for more than a decade: the patient remains tumor-free for over 8 years, showing an exceptionally favorable course of the disease, and thus could be considered as cured under comparable circumstances in case of many other malignancies. Against this background, we consider it worthwhile to reassess this case after this extended observation time, aiming to provide additional in-depth T-cell immune monitoring results.

## Results

### Clinical course and treatment

**Figure 1:**
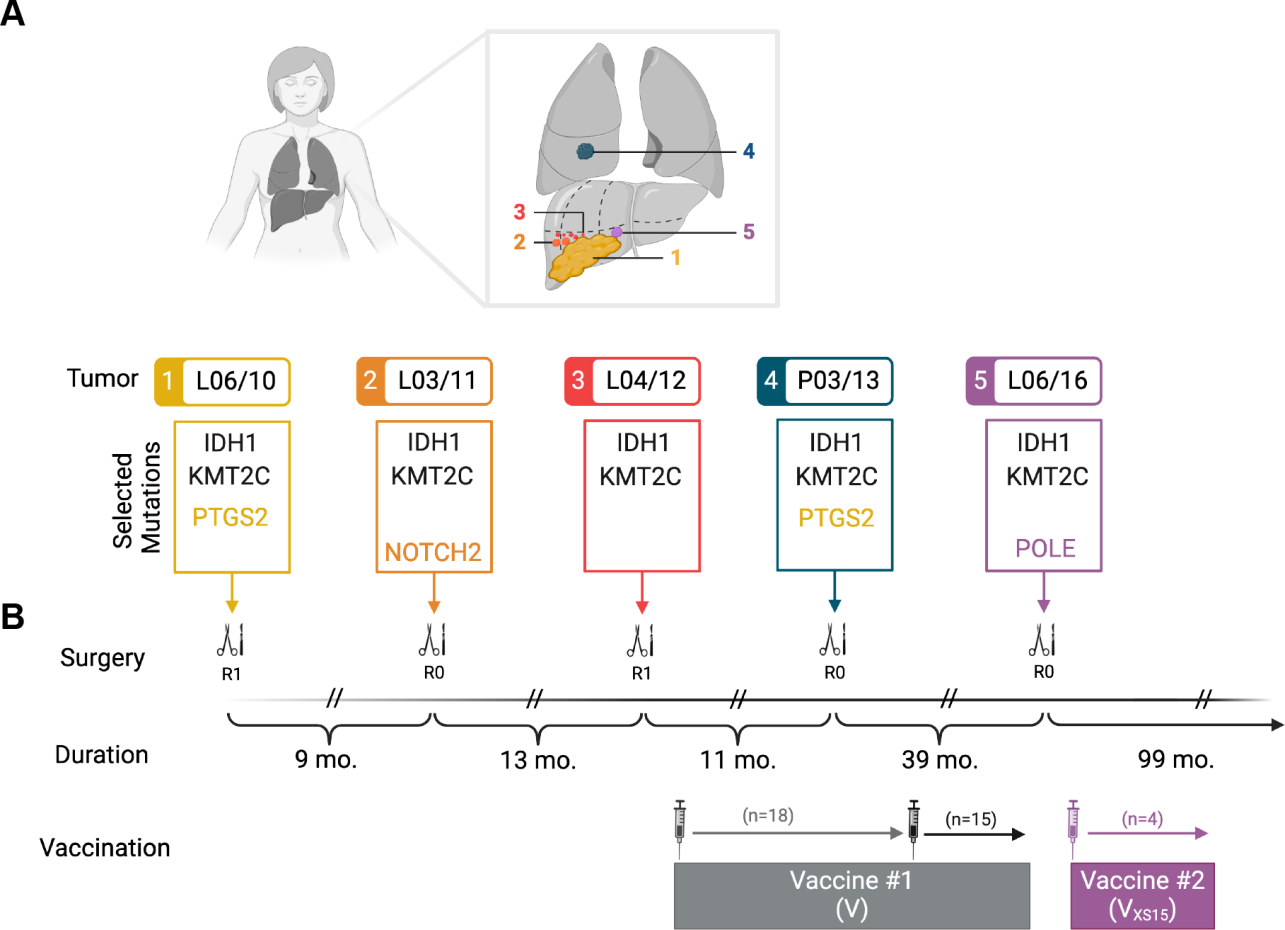
Timeline of events and tumors’ characteristics. (**A**) Schematic view of the primary tumor location and the successive recurrences (tumor coding L: liver, P: pulmonary, month/year) as well as corresponding shared and private oncogenic variants that were chosen to be assessed by T-cell immune monitoring. (**B**) Further key events, including surgical interventions (R0/R1) and administration of personalized peptide vaccines, are depicted in a timeline with annotated durations in months (mo.) as well as number of vaccinations. Created in BioRender. https://BioRender.com/. IDH1, isocitrate dehydrogenase 1; KMT2C, lysine methyltransferase 2C; NOTCH2, notch receptor 2; POLE, DNA polymerase epsilon, catalytic subunit; PTGS2, prostaglandin-endoperoxide synthase 2; R0, no residual tumor; R1, microscopic residual tumor.

In 2010, a patient in her mid-50s was incidentally diagnosed with a large iCCA (poorly differentiated (G3) adenocarcinoma: 11.5 cm in diameter involving segments IVb/V/VI) and lymph node metastasis (L06/10). The tumor (pT1, pN1 (1/20), L0, V0, G3, R1, M0 according to the *Union Internationale Contre le Cancer* (UICC) staging system) was resected but subsequently recurred in the liver 9 months (L03/11 in segments V/VI) and 22 months (L04/12 requiring right hemihepatectomy) as well as in the lungs 33 months (P03/13 affecting middle lobe and arteria pulmonalis) and once again in the liver (L06/16 affecting segment IV and hepatic fork) 72 months after initial diagnosis (figure 1A). Treatment involved resection of the tumor or metastases in every instance as well as vaccination with two individualized vaccines (V and V_XS15_), each containing seven tumor-associated peptides (TUMAPs) (online supplemental table S1A,B) administered as an experimental treatment attempt (figure 1B). On a side note, the patient was never treated with ICB in the course of the treatment described here. The clinical course and immune monitoring findings after treatment with the first vaccine (V) as well as details regarding her cancer were previously published, shortly preceding her last tumor recurrence (L06/16).^6^ Additionally, the same shared oncogenic drivers were identified in all preceding tumors and in the last occurring lesion (*IDH1* NM_001282386.1:c.394C>T, *KMT2C* NM_170606.2:c.12545A>G), while a possibly subclonal variant (*POLE* NM_006231.3:c.772A>C) was detected in L06/16 as well as in L03/11 (*NOTCH2* NM_024408.3:c.3416T>G) (figure 1A).

### Tumor-infiltrating lymphocytes recognize vaccine peptides

**Figure 2:**
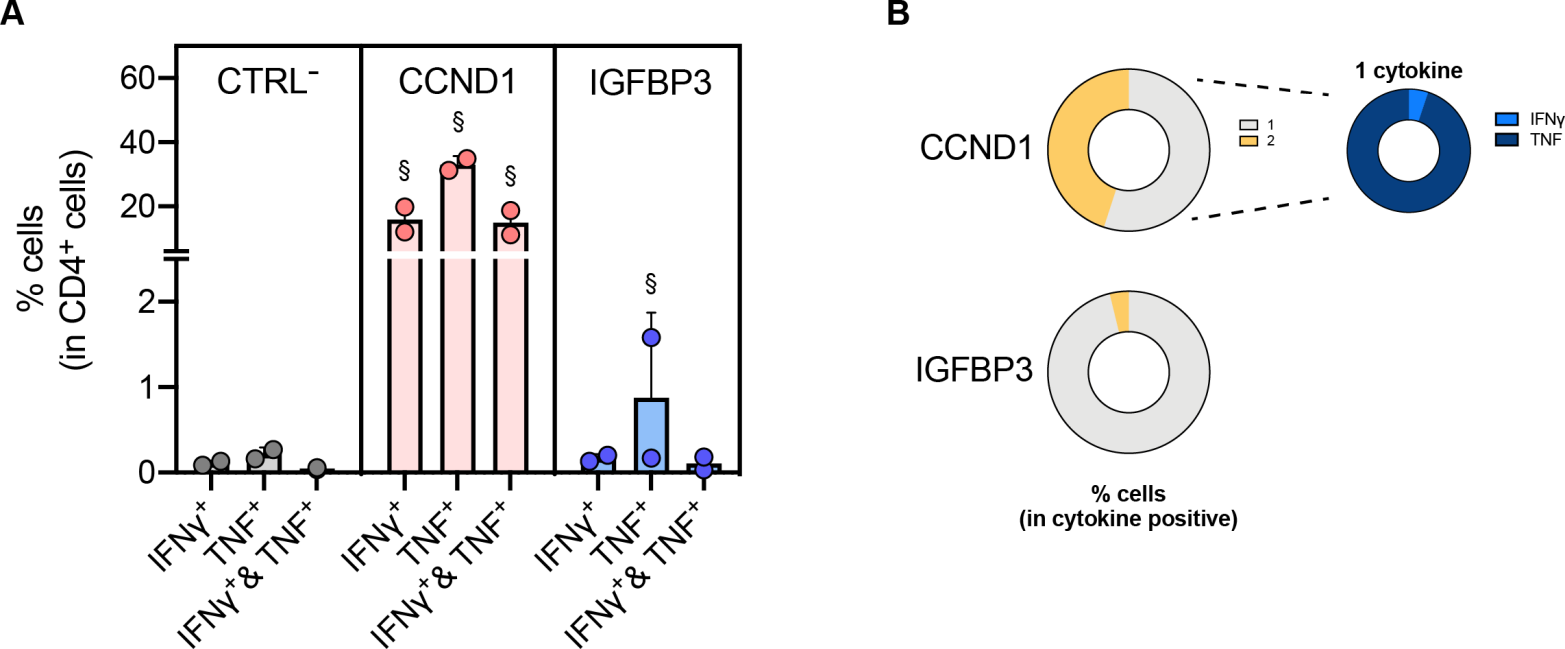
Vaccine (V)-specific T cells in TILs. (**A**) Quantification of the % of cytokine-producing cells in TILs (L06/16, collected between V33 (first vaccine) and Pre V_XS15_ (second vaccine)). Expanded TILs were cultured for 12 days *in vitro* in the presence of the vaccine TUMAPs and interleukin-2. Afterwards, cells were re-incubated for 14 hours with the peptides and the cytokine production assessed by intracellular cytokine staining. Graph shows the % of CD4^+^ cells that produce IFN-γ, TNF or both cytokines (IFN-γ and TNF). DMSO was used as a negative control (CTRL^−^). Each dot represents one tumor piece analyzed from which TILs were derived (n=2), bars represent means, and § mark conditions that were considered positive (see the Materials and Methods provided in the online supplemental file). (**B**) Percentage of CD4^+^ cells that produce one (gray color) or two cytokines (yellow color) within cytokine-producing cells is shown (average of both tumor pieces). On the right panel, the distribution of cells that produce either IFN-γ or TNF within single cytokine-producing cells is shown after re-stimulation with CCND1. CCND1, cyclin D1; CTRL^-^, negative control; DMSO, dimethyl sulfoxide; IFN, interferon; IGFBP3, insulin-like growth factor-binding protein 3; TIL, tumor-infiltrating lymphocytes; TNF, tumor necrosis factor; TUMAP, tumor-associated peptide.

The resected recurrent liver lesion (L06/16) was processed *in vitro* and isolated tumor-infiltrating lymphocytes (TILs) underwent a 12-day expansion protocol in the presence of the first vaccine TUMAPs (online supplemental table S1A) and interleukin-2. On re-stimulation with the human leukocyte antigen (HLA) class II peptide from cyclin D1 (CCND1), tumor necrosis factor (TNF) production was readily observed within a large fraction of CD4^+^ cells (approximately 30%), with half of them simultaneously producing interferon (IFN)-γ (figure 2A,B and online supplemental figure S1 for gating and exemplary dot-plots). Insulin-like growth factor-binding protein 3 (IGFBP3)-reactive CD4^+^ T cells were also detected, although to a lower extent (<2% TNF^+^ CD4^+^ cells) and with negligible multifunctionality (figure 2A,B). Despite the previous detection of matrix metalloproteinase-7 (MMP7)-specific and regulator of G-protein signaling 5 (RGS-5)-specific T cells in the peripheral blood of the patient on vaccination, reactivity of the TILs against these or any of the other HLA class I TUMAPs included in the first vaccine (online supplemental table S1A) was not observed (data not shown).

Altogether, these results demonstrate that vaccine-induced CD4^+^ T cells reached the tumor tissue and are functionally active when tested *in vitro*.

### The tumor invasive front is characterized by a dense immune infiltrate and stains positive for HLA-DR

Immunohistochemical analyses of the tumor microenvironment further showed a dense immune infiltrate at the invasive front of L06/16 (online supplemental figure S2), characterized by both CD4^+^ and CD8^+^ cells as well as macrophages. In contrast to previous tumor manifestations (online supplemental figure S3–S6), this immune infiltrate appears to be more structured with CD3^+^ cells giving the impression of surrounding the tumor cells as a first line and macrophages located behind them. Furthermore, stainings for programmed death-ligand 1 (PD-L1) showed single positive cells within the tumor and FOXP3 revealed faint focal staining in only a few immune cells, which can be observed in L06/16 (online supplemental figure S7) as well as in previous tumor manifestations (online supplemental figures S8–S10), suggesting that neither regulatory T cells nor the programmed cell death protein 1 (PD-1)/PD-L1 axis are prominent immune suppressive factors. In contrast, HLA-DR is observed abundantly in peritumoral areas as well as interspersed inside the tumor itself. Macrophages are likely accounting for a substantial fraction of this HLA-DR staining, while additional cells, particularly some located at the invasive front, do also stain positive for HLA-DR, but remain negative for macrophage markers. However, the majority of the cancer cells themselves appears to be negative for HLA-DR.

### Personalized peptide vaccination induces durable and functional T-cell responses that can be boosted and expanded by novel peptides and adjuvants

**Figure 3:**
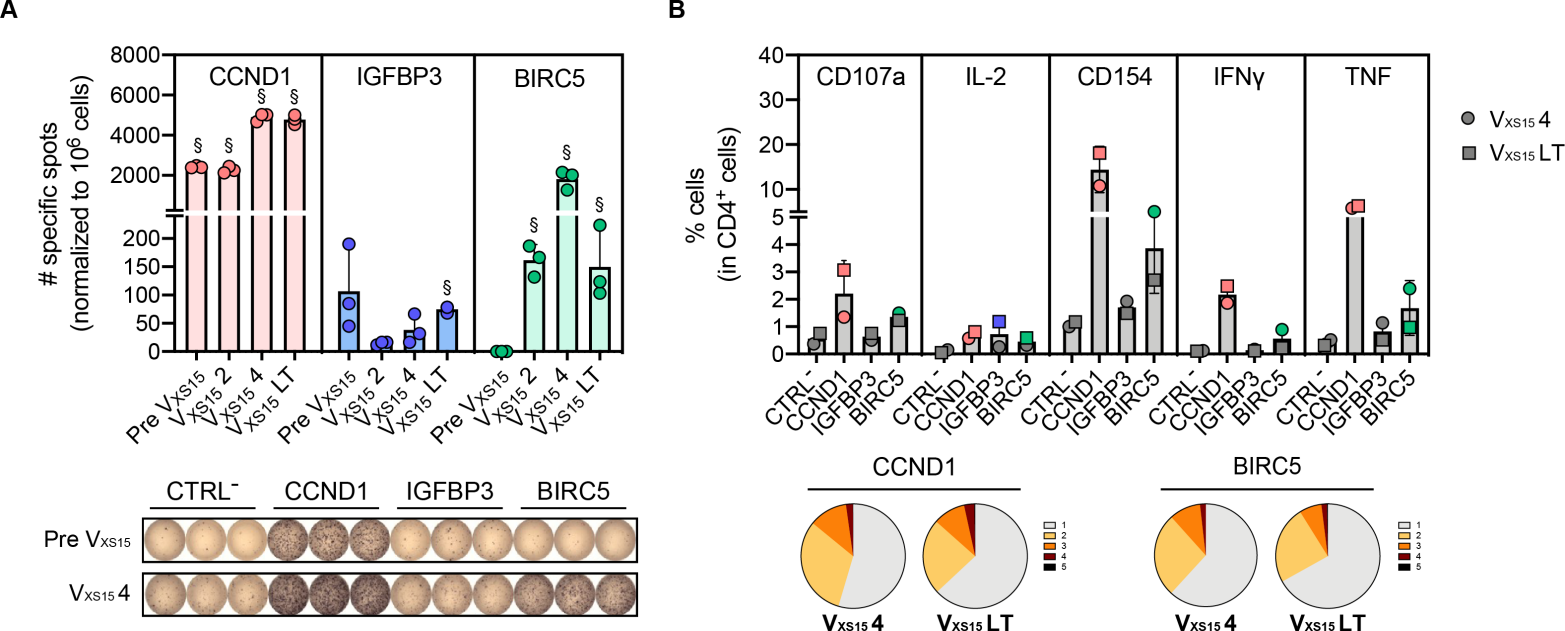
T-cell responses to HLA class II peptides of the second vaccine (V_xs15_). (**A**) Quantification and representative wells of the IFN-γ ELISpot with HLA class II TUMAPs (CCND1, IGFBP3, BIRC5). PBMCs were stimulated *in vitro* for 12 days in the presence of the vaccine peptides prior to IFN-γ ELISpot analyses. Graph shows specific spots per 10^6^ cells (background (CTRL^−^) subtracted) prior to the second vaccination (Pre V_xs15_), after 2 (V_xs15_ 2) and 4 (V_xs15_ 4) vaccinations, and in the long term (V_XS15_ LT, 5 years after last vaccination). § Marks conditions that were considered positive (see Materials and Methods provided in the online supplemental file). Each condition was investigated in triplicates (each well is shown as a dot in the graph and bars represent means). (**B**) Quantification of activation marker-producing cells. After a 12-day *in vitro* stimulation, cells (after four vaccinations (V_XS15_ 4, circle symbols) or long-term (V_XS15_ LT, square symbols)) were re-stimulated overnight with the peptides and their functionality investigated by intracellular cytokine staining. Bar graph shows the mean % activation-positive marker within CD4^+^ T cells. Colored symbols show conditions that were considered positive, while grey symbols indicate conditions that were regarded as negative, according to the positivity criteria used (see Materials and Methods). For CCND1-, IGFBP3- and BIRC5-derived TUMAPs, symbols are shown in pink, blue and green, respectively. Multifunctionality analyses of cells that produce either one (gray), two (yellow), three (orange), four (dark red) or five (black) markers are shown as pie charts on the lower panel for CCND1 and BIRC5. BIRC5, baculoviral inhibitor of apoptosis repeat containing 5 (survivin); CCND1, cyclin D1; CTRL^-^, negative control; ELISpot, enzyme-linked immunosorbent spot assay; HLA, human leukocyte antigen; IGFBP3, insulin-like growth factor-binding protein 3; IFN, interferon; IL, interleukin; LT, long-term; PBMCs, peripheral blood mononuclear cells; TNF, tumor necrosis factor; TUMAP, tumor-associated peptide.

Based on the molecular reassessment of the new tumor lesion L06/16, including immunopeptidomics, a second peptide vaccine (V_XS15_) was designed. This new vaccine contained eight HLA class I/II peptides including seven TUMAPs, comprising only peptides that show the required properties for presentation on the patients’ own HLAs (see online supplemental table S1B). Peptides previously shown to be immunogenic^6^ were kept from the first vaccine and two novel TUMAPs derived from cancer-testis antigens with immunopeptidome evidence were selected, as well as one survivin (BIRC5 (baculoviral inhibitor of apoptosis repeat containing 5))-derived peptide (see Materials and Methods provided in the online supplemental file). Moreover, the novel adjuvant XS15 (a toll-like receptor (TLR)1/2 agonist)^7^ was used. A strong T-cell response to CCND1 that was further boosted after four vaccinations with the new vaccine was seen (figure 3A). In contrast, the IGFBP3 peptide was only weakly recognized (figure 3A), in accordance with the observations from the TILs (figure 2). The novel HLA class II TUMAP BIRC5 also drove the production of IFN-γ on stimulation of the peripheral blood mononuclear cells (PBMCs). This reactivity appeared after two vaccinations and increased with further vaccinations (figure 3A). CCND1-reactive and BIRC5-reactive CD4^+^ T cells were highly functional, with 25–40% of the *in vitro* expanded cells producing at least two of the investigated activation markers (figure 3B and online supplemental figure S11A–C). Particularly, CCDN1 peptide stimulation led to the upregulation of the degranulation marker CD107a, which might indicate that reactive CD4^+^ cells comprise cytotoxic effectors. Strikingly, vaccine-specific T cells persisted in the blood of the patient and could be detected 5 years after the administration of the last vaccination (figure 3A).

In contrast to this strong reactivity against two out of three HLA class II peptides, TUMAP-specific T cells against the HLA class I peptides included in the second vaccine (V_XS15_) could not be detected, including against the RGS-5 and MMP7 peptides that were contained in the previous vaccine (V) and were shown at earlier time points to be recognized.^6^ Only a weak reactivity against the novel reporter Cytomegalovirus (CMV) sequence was observed (online supplemental figure S12). Hence, anti-vaccine CD4^+^ T cells could be induced by the new multi-peptide vaccine adjuvanted with XS15 and were sustained in the long term.

### Functional T cells recognizing tumor-specific neoantigens are detectable in the blood and show long-term persistence

**Figure 4:**
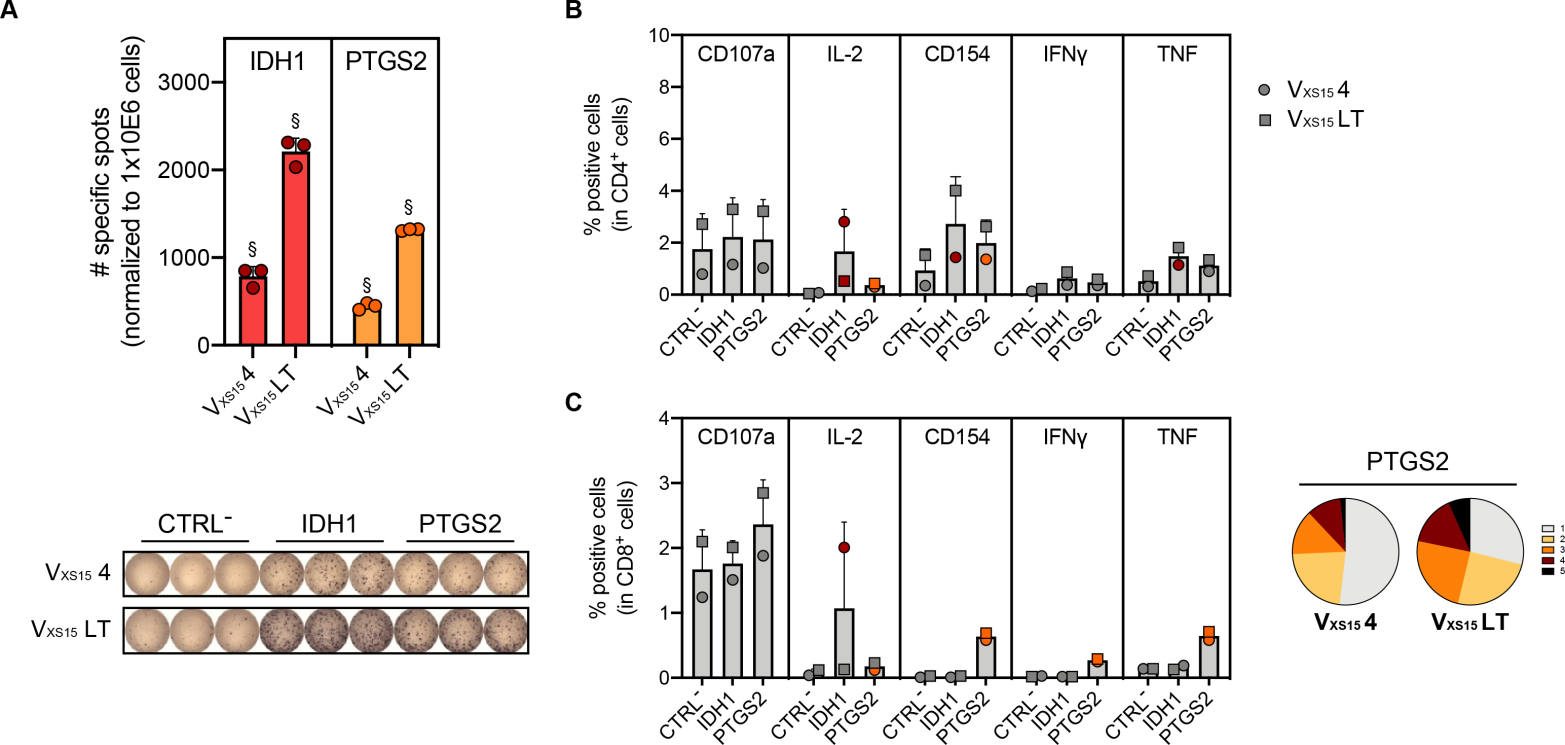
T-cell responses to tumor-specific gene variants. (**A**) Quantification and representative wells of the IFN-γ ELISpot with variant-derived peptides (IDH1_R132C_, red color; PTGS2_V102L_, orange color). PBMCs were stimulated *in vitro* with the peptides for 12 days prior to IFN-γ ELISpot analyses. Graph shows specific spots per 10^6^ cells (background (CTRL^-^) subtracted) for two different time points (V_XS15_ 4, after four vaccinations) and in the long term (V_XS15_ LT, 5 years after last vaccination). § Marks conditions that were considered positive (see Materials and Methods provided in the online supplemental file). Each condition was investigated in triplicates (each well is shown as a dot and bars represent means). (**B and C**) Quantification of activation marker-producing cells in CD4^+^ (**B**) and CD8^+^ (**C**) cells. After a 12-day *in vitro* stimulation, cells (after four vaccinations (circle symbols) or long-term (square symbols)) were re-stimulated overnight with the peptides and their activation investigated by intracellular cytokine staining. Bar graphs show mean % of activation-positive marker. Colored symbols show conditions that were considered positive, while grey symbols indicate conditions that were regarded as negative, based on the positivity criteria used (see Materials and Methods). For IDH1- and PTGS2-derived TUMAPs, colored symbols are shown in red and orange, respectively. Multifunctionality analyses showing the % of activation marker positive cells that produce either one (gray), two (yellow), three (orange), four (dark red) or five (black) markers is shown on the right panel for PTGS2_V102L_ for CD8^+^ cells (**C**). CTRL^-^, negative control; ELISpot, enzyme-linked immunosorbent spot assay; IDH1, isocitrate dehydrogenase 1; IFN, interferon; IL, interleukin; LT, long-term; PBMCs, peripheral blood mononuclear cells; PTGS2, prostaglandin-endoperoxide synthase 2; TNF, tumor necrosis factor; TUMAP, tumor-associated peptide.

Detailed molecular analyses of the tumor samples collected during surgical resection^6^ (figure 1A) revealed several stable (i.e., in *IDH1*, *KMT2C*) or tumor sample-specific (i.e., in *PTGS2*, *POLE*, *NOTCH2*) genetic variants. Using 20 mer synthetic peptides, we tested whether these five selected genetic variants (online supplemental table S1C) might have induced a natural T-cell response. Indeed, T-cell reactivity against IDH1_R132C_ and PTGS2_V102L_ was detected in circulation at multiple time points prior to the application of the second vaccine (online supplemental figure S13A) and was still evidenced on resection of the tumor lesion L06/16 (figure 4, online supplemental figure S13B,C for representative dot-plots). Not only was IFN-γ secretion detected on stimulation of the patient PBMCs with the mutation-derived peptides (figure 4A), but further investigation showed that IDH1_R132C_ was recognized by CD4^+^ T cells and PTGS2_V102L_ by both CD4^+^ and CD8^+^ T cells (figure 4B,C). Importantly, approximately 50–70% of these CD8^+^ T cells were multifunctional (figure 4C). These results demonstrate spontaneous and long-lasting T-cell recognition of one shared (IDH1_R132C_) and one private (PTGS2_V102L_) tumor genetic variant.

## Discussion

We report here on the treatment course and follow-up of a patient over more than a decade, who was diagnosed with a metastatic CCA and received two different successive personalized multi-peptide cancer vaccines, both based on non-mutated peptides. Extraordinarily, the patient has recently completed a follow-up CT scan (11/2024) and remains disease-free more than 8 years after her last relapse and 14 years after initial diagnosis.

In the course of her disease, the patient received multiple injections of a first peptide vaccine, with details described previously.^6^ The disease was controlled for 3 years, then the patient experienced a novel local relapse. The prior favorable clinical course and a novel molecular analysis of the relapsed tumor prompted us to design an adapted multi-peptide vaccine where four previously immunogenic TUMAPs were kept, and three novel ones were included. This second vaccine was administered four times throughout approximately 2 years following the last surgery, together with a novel potent TLR1/2 ligand as an adjuvant.^7^ Overall, *in vitro* assessment of vaccine immunogenicity showed predominantly CD4^+^ T cell-mediated responses. In particular, the strong reactivity against the CCND1 peptide, which had been included in the first vaccine, persisted for more than 10 years, showing establishment of long-term T-cell memory. In-depth analysis also demonstrated multifunctionality on activation, including the expression of the degranulation marker CD107a in a subset of CCND1-specific cells. We speculate that such CD4^+^ T cells possess cytotoxic potential as it has previously been described;^8^,^10^ however, the capacity of these cells to directly kill tumor cells *in vitro* could not be assessed. Notably, we did neither observe substantial HLA-DR staining of tumor cells nor staining of PD-L1, while both CD4^+^ and HLA-DR^+^ cells could be abundantly shown within the tumor microenvironment, with scarce FOXP3^+^ cells. These findings, while questioning a prominent direct tumor recognition through HLA class II-presented peptides by CD4^+^ T cells, de-emphasize key immunosuppressive mechanisms and suggest alternative effector mechanisms mediated by CD4^+^ T cells.

Importantly, TIL analysis clearly demonstrated that CCND1-specific cells had the ability to migrate into the tumor site. Apart from this dominant anti-CCND1 response, CD4^+^ T-cell reactivities against BIRC5 (survivin) and to a much lower extent IGFBP3 were also detected in the blood and/or tumor tissue. Finally, we could also demonstrate that two (most probably initiating) truncal oncogenic gene variants in *IDH1* (very frequently affected by mutations in iCCAs) and *PTGS2* were spontaneously recognized by CD4^+^ (both variants) and even CD8^+^ (PTGS2 only) T cells that were detectable over almost the entire observation period.

Overall, the CD4^+^ T-cell responses that we observed were superior to CD8^+^ T-cell responses both in terms of strength and persistence over time, which has also been reported in recent vaccine studies employing synthetic peptides or messenger RNA targeting either non-mutated or neoantigen-derived sequences.^11^,^13^ Various factors may explain the failure of the vaccine to induce strong CD8^+^ T-cell responses, including *inter alia* the choice of the vaccine peptides themselves, the nature of the adjuvant (although excellent CD8^+^ T-cell priming with XS15 was demonstrated before^7^), or a more general faint responsiveness of the CD8^+^ T-cell compartment in this particular patient. Still, these observations are in line with the increasing number of reports that demonstrate the importance and pleiotropic role of CD4^+^ T cells in antitumor T-cell responses, including studies showing their capacity to directly kill tumor cells.^8^,^10^ Although we wish to explicitly state that there is a possibility that the patient presented here may constitute an extreme outlier with distinct tumor biology and atypical features and therefore generalizability should not be assumed, it is tempting to speculate that both vaccine-induced and naturally occurring tumor neoantigen-specific CD4^+^ T-cell responses played a key role in tumor immunosurveillance and have jointly contributed to the exceptionally favorable clinical course of this patient.

## Supplementary material

10.1136/jitc-2025-012107 online supplemental file 1

## References

[1] Greten TF, Schwabe R, Bardeesy N (2023). Immunology and immunotherapy of cholangiocarcinoma. Nat Rev Gastroenterol Hepatol.

[2] Mavros MN, Economopoulos KP, Alexiou VG (2014). Treatment and Prognosis for Patients With Intrahepatic Cholangiocarcinoma: Systematic Review and Meta-analysis. JAMA Surg.

[3] Tran E, Turcotte S, Gros A (2014). Cancer immunotherapy based on mutation-specific CD4+ T cells in a patient with epithelial cancer. Science.

[4] Manthopoulou E, Ramai D, Dhar J (2023). Cholangiocarcinoma in the Era of Immunotherapy. *Vaccines (Basel*).

[5] Halder R, Amaraneni A, Shroff RT (2022). Cholangiocarcinoma: a review of the literature and future directions in therapy. Hepatobiliary Surg Nutr.

[6] Löffler MW, Chandran PA, Laske K (2016). Personalized peptide vaccine-induced immune response associated with long-term survival of a metastatic cholangiocarcinoma patient. J Hepatol.

[7] Rammensee H-G, Wiesmüller K-H, Chandran PA (2019). A new synthetic toll-like receptor 1/2 ligand is an efficient adjuvant for peptide vaccination in a human volunteer. J Immunother Cancer.

[8] Oh DY, Kwek SS, Raju SS (2020). Intratumoral CD4^+^ T Cells Mediate Anti-tumor Cytotoxicity in Human Bladder Cancer. Cell.

[9] Richardson JR, Schöllhorn A, Gouttefangeas C (2021). CD4+ T Cells: Multitasking Cells in the Duty of Cancer Immunotherapy. Cancers (Basel).

[10] Cachot A, Bilous M, Liu Y-C (2021). Tumor-specific cytolytic CD4 T cells mediate immunity against human cancer. Sci Adv.

[11] Hilf N, Kuttruff-Coqui S, Frenzel K (2019). Actively personalized vaccination trial for newly diagnosed glioblastoma. Nature.

[12] Sahin U, Derhovanessian E, Miller M (2017). Personalized RNA mutanome vaccines mobilize poly-specific therapeutic immunity against cancer. Nature.

[13] Slingluff CL, Petroni GR, Chianese-Bullock KA (2011). Randomized multicenter trial of the effects of melanoma-associated helper peptides and cyclophosphamide on the immunogenicity of a multipeptide melanoma vaccine. J Clin Oncol.

