# Supplementary material for: Exceptional tumor-free survival of a patient with metastatic intrahepatic cholangiocarcinoma after surgery and personalized peptide vaccination: revisiting a striking case
Source: J Immunother Cancer. 2025 Oct 9;13(10):e012107. doi: 10.1136/jitc-2025-012107 (PMC13059883; doi:10.1136/jitc-2025-012107)
Supplement: online supplemental file 1 [file jitc-13-10-s001.pdf]

Supplement to:

Exceptional tumor-free survival of a patient with metastatic intrahepatic cholangiocarcinoma after surgery and personalized peptide vaccination: revisiting a striking case

Ana Maia [1], Juliane Schuhmacher [1], Silvio Nadalin [2], Alfred Königsrainer [2,3], Karolin Thiel [2,4], Annika Nelde [3,5], Raphael S. Zinser [1], Christopher Schroeder [6,7], Sven Mattern [8], Stephan Singer [8], Hans Bösmüller [8], Hans-Georg Rammensee [1,3,7], Markus W. Löffler [1,3,7,9] and Cécile Gouttefangeas [1,3,7]

[1] Institute of Immunology, University and University Hospital Tübingen, Tübingen, Germany

[2] Department of General, Visceral and Transplant Surgery, University Hospital Tübingen, Tübingen, Germany

[3] Cluster of Excellence iFIT (EXC2180) 'Image-Guided and Functionally Instructed Tumor Therapies', University of Tübingen, Tübingen, Germany

[4] Department of General, Visceral and Thoracic Surgery, Oberschwabenklinik gGmbH, St. Elisabethen-Klinikum, Ravensburg, Germany

[5] Institute of Immunology, Department of Peptide-based Immunotherapy, University and University Hospital Tübingen, Tübingen, Germany

[6] Institute of Medical Genetics and Applied Genomics, University Hospital Tübingen, Tübingen, Germany

[7] German Cancer Consortium (DKTK) and German Cancer Research Center (DKFZ), partner site Tübingen, Heidelberg, Germany

[8] Institute of Pathology and Neuropathology, University Hospital Tübingen, Tübingen, Germany

[9] Institute for Clinical and Experimental Transfusion Medicine (IKET), University Hospital Tübingen, Tübingen, Germany

Table of contents

Supplementary materials and methods **3-8**

Supplementary Table S1: Synthetic peptides **9**

Supplementary Table S2: mAbs panels for ICS **10**

Supplementary Table S3: Abs used for IHC **10**

Supplementary Figure S1: Vaccine (V)-specific T cells in TILs **11**

Supplementary Figure S2: H&E and IHC (CD4, CD8, CD3, CD163) staining of recurrent liver tumor (L06/16) **12**

Supplementary Figure S3: H&E and IHC (CD4, CD8, CD3, CD163) staining of pulmonary metastasis (P03/13) **13**

Supplementary Figure S4: H&E and IHC (CD4, CD8, CD3, CD163) staining of recurrent liver tumor (L04/12) **14**

Supplementary Figure S5: H&E and IHC (CD4, CD8, CD3, CD163) staining of recurrent liver tumor (L03/11) **15**

Supplementary Figure S6: H&E and IHC (CD4, CD8, CD3, CD163) staining of primary liver tumor (L06/10) **16**

Supplementary Figure S7: H&E and IHC (CD68, FOXP3, HLA-DR, PD-L1) staining of recurrent liver tumor (L06/16) **17**

Supplementary Figure S8: H&E and IHC (CD68, FOXP3, HLA-DR, PD-L1) staining of recurrent liver tumor (L04/12) **18**

Supplementary Figure S9: H&E and IHC (CD68, FOXP3, HLA-DR, PD-L1) staining of recurrent liver tumor (L03/11) **19**

Supplementary Figure S10: H&E and IHC (CD68, FOXP3, HLA-DR, PD-L1) staining of primary liver tumor (L06/10) **20**

Supplementary Figure S11: T cell responses to HLA class II peptides after application of the 2nd vaccine **21**

Supplementary Figure S12: T cell response to HLA class I peptides of the 2nd vaccine **22**

Supplementary Figure S13: T cell responses to mutated peptides **23**

References **24-25**

Supplementary materials and methods

Biomaterial and ethics statement

Patient treatment was initiated in 09/2012 after comprehensive and detailed informed consent by multiple attending physicians and a written approval provided by the patient as part of a research project (IndividuaLIVER, Project Nr. 180/2011BO2; approved by the Institutional Review Board at the University Hospital of Tübingen). Informed consent was obtained also for all subsequent treatments outside of clinical routines, providing sufficient reflection periods in each case. Patient treatment was performed according to applicable national law and regulations (cf. statement WD 9 – 3000 – 083/23 of the German Parliament) and applicable guidelines (Declaration of Helsinki, 8th Revision 2008 in Seoul, § 35) for which Institutional Review Board or ethics committee review is not required. The patient has been duly informed and has explicitly consented to all non-standard treatment attempts and informed consent was obtained in each case before any such treatment by the treating physicians. Biomaterials were handled according to the statutes of the local biobank, approved by the Institutional Review Board (Project Nr. 501/2013BO2). The patient´s HLA-typing was determined as HLA-A*03:01/*29:01, -B*07:05/*35:01, -C*04:01/*15:05, -DRB1*01:01/*11:01, -DRB3*02:02, -DQB1*03:01/*05:01 [10]. Written informed consent was obtained from the patient for publication of this case report including medical details and any accompanying materials.

Next Generation Sequencing and Data Analysis

For sequencing the recurrent tumor L06/16, hybridization-based custom cancer panel, which includes 336 genes was used for enrichment (SureSelect XT; Agilent, Santa Clara, CA, USA) and the library sequenced (NextSeq or MiSeq, Illumina, San Diego, CA, USA). A sequencing depth of 815x was achieved for the tumor sample and 705x for the normal tissue reference. Sequencing data were analyzed with our in-house megSAP pipeline (https://github.com/imgag/megSAP doi: 10.5281/zenodo.13744183). SeqPurge [1] was used for adapter trimming, samblaster for deduplication [2] and bwa mem (v 0.7.15, https://github.com/lh3/bwa) for mapping against hg19. Structural variants were called with manta [3], CNVs detected using cnvhunter (https://github.com/imgag/ngs-bits) and small variants called with strelka [4]. Variant annotation was done with SnpSift and SnpEff [5] and annotated using in-house and public databases**.**

Immunopeptidomics

HLA class I and HLA class II molecules were isolated from lysates of tissue samples of lesion L06/16 by standard immunoaffinity purification [6] using the pan–HLA class I-specific W6/32, the pan-HLA class II-specific Tü-39, and the HLA-DR-specific L243 monoclonal antibodies (produced in house) cross-linked to CNBr-activated Sepharose (Sigma-Aldrich, Burlington, MA, USA). Peptides were eluted by 0.2% trifluoroacetic acid, isolated by ultrafiltration (Amicon filter units; Merck Millipore, Burlington, MA, USA), lyophilized, and desalted using ZipTip pipette tips with C18 resin (Merck Millipore).

For the mass spectrometric analysis, peptides were separated by nanoflow high-performance liquid chromatography (RSLCnano, Thermo Fisher Scientific) using a 50 µm × 25 cm PepMap rapid separation column (Thermo Fisher Scientific) and a linear gradient ranging from 2.4% to 32.0% acetonitrile at a flow rate of 0.3 µl/minute over the course of 90 minutes. Eluting peptides were analysed in an online-coupled Orbitrap Fusion Lumos mass spectrometer (Thermo Fisher Scientific) equipped with a nanoelectron spray ion source using a data dependent acquisition mode using a top speed collisional-induced dissociation (CID, normalized collision energy 35%, HLA class I peptides) or higher-energy collisional dissociation (HCD, normalized collision energy 30%, HLA class II peptides) fragmentation method. Mass range for HLA class I peptide analysis was set to 400–650 m/z with charge states 2+ and 3+ selected for fragmentation. For HLA class II peptide analysis, mass range was limited to 400–1,000 m/z with charge states 2+ to 5+ selected for fragmentation.

For data processing, the SEQUEST HT search engine (University of Washington, Seattle, WA) [7] was used to search the human proteome as comprised in the Swiss-Prot database (September 27, 2013) without enzymatic restriction. Precursor mass tolerance was set to 5 ppm, and fragment mass tolerance to 0.02 Da. Oxidized methionine was allowed as a dynamic modification. The FDR was estimated using the Percolator algorithm [8] and limited to 1%. Peptide lengths were limited to 8 to 12 amino acids for HLA class I and to 8 to 25 amino acids for HLA class II.

The mass spectrometry immunopeptidomics data generated in this study have been deposited to the ProteomeXchange Consortium (http://proteomecentral.proteomexchange.org) via the PRIDE [9] database partner repository with the dataset identifier PXD059585.

Vaccine design and formulation

Vaccine design was based on immune monitoring T cell data performed following multiple vaccinations of the patient with the 1st vaccine, as well as on next generation sequencing, histopathology and immunopeptidomics data as published previously [10] and provided in this report. A multitudinous selection committee consisting of experienced clinicians, immunologists and biochemists convened to optimize the vaccine formulation after the tumor relapse L06/16. Based on the immune responses evidenced already and the generally favorable clinical course of the patient, it was decided to retain HLA class I-binding peptides GLASFKSFLK (derived from RGS-5) and SLFPNSPKWTSK (derived from MMP7) as well as HLA class II-binding peptides NPPSMVAAGSVVAAV (derived from CCND1) and HSKIIIIKKGHAKDSQ (derived form IGFBP3). The CMV pp65-derived marker peptide TPRVTGGGAM [11] was also added, a strategy used also in various other vaccine studies [12].

In addition, two peptides with a clear cancer/testis antigen profile (see https://www.proteinatlas.org/) and with immunopeptidomic evidence were additionally chosen (FVQENYLEY derived from MAGEA3/A6 and SPIDPSEKY derived from CATSPERG). Ultimately, the highly immunogenic promiscuous survivin peptide TLGEFLKLDRERAKN (derived from BIRC5) was included [13].

For adjuvantation and based on preliminary data from self-experimentation [14], the novel adjuvant XS15 (TLR1/2 ligand) at a concentration of 37,5 µg/vaccination was added to 225 µg of each of the eight peptides mentioned above, using 25% dimethyl sulfoxide (DMSO) in water at a total volume of 500µl. The vaccine was emulsified by mixing with montanide ™ ISA51 VG (Seppic, Paris, France) 1:1 to obtain a homogeneous water-in-oil emulsion and injecting this vaccine subcutaneously (*s.c.)* at the abdomen.

Peptide production

Peptides were synthesized in-house (Institute for Immunology, University of Tübingen, Germany) by solid-phase synthesis using the 9-fluorenylmethyl-oxycarbonyl/tert-butyl (Fmoc/tBu) strategy [15] with an automated peptide synthesizer (EPS 221, Abimed; ABI 433A, Applied Biosystems). Lyophilized peptides were diluted at 1 mg/ml in MilliQ water with 10% DMSO, aliquoted and stored at −80°C. The mutated 20mers were designed so that predicted embedded HLA class I (HLA-A*03, -B*07 and -B*35) binders would be at the C-terminal position, based on our previous observations that HLA class I epitopes elongated at the N-terminal end are better recognized [16]. All peptides are listed in **Suppl. Tables S1A-C** and

abbreviated with their corresponding source protein names. For mutated sequences, the position and the exchanged amino acids are given.

PBMCs and TILs isolation and expansion

EDTA-anticoagulated blood (45 ml) was drawn before vaccination with the second vaccine (**Suppl. Table S1B**), at regular intervals during the course of vaccination, and post-vaccination. PBMCs were isolated by density gradient centrifugation, frozen and stored in liquid nitrogen, essentially as previously described [10].

Fresh tissue obtained during resection of the reoccurring CCA L06/16 was taken for isolation of tumor infiltrating lymphocytes (TILs). Briefly, tissue was transferred into culture medium immediately after surgery and further processed under sterile conditions. Tissue was rinsed in cold PBS, then cut into small fragments (1-2 mm^3) and up to four fragments were placed in one well of a 24 well plate. A total of 2 ml of TIL medium (Iscove's modified Dulbecco's medium (IMDM) supplemented with 7.5% heat-inactivated human AB serum (hi HS) and 1% penicillin/streptomycin (PenStrep) containing 1 µg/ml HLA class I peptides or 5 µg/ml HLA class II peptides contained in the first vaccine (**Suppl. Table S1A**) and 1000 IU/ml of IL-2 (PROLEUKIN®) was added to the tumor pieces. Cultures were placed at 37°C and 7.5% CO_2. Medium was refreshed every 2-3 days by removing 1 ml from each well and adding 1 ml of fresh medium supplemented with IL-2 to a final concentration of 1000 IU/ml. On day 12, cultures were collected, cells were counted using Trypan blue then directly used for T cell assays.

T cell analyses

In vitro stimulation (IVS)

Before testing, PBMCs were expanded *in vitro* as previously described [10,17]. Briefly, PBMCs were thawed in thawing medium (IMDM supplemented with 2.5% hi HS, 1% penicillin / streptomycin, 50 µM beta-mercaptoethanol (β-ME) and 3 µg/ml DNase I), washed (1300 rpm, 8 min, RT), counted, resuspended in T cell medium (TCM – IMDM supplemented with 10% hi HS, 1% PenStrep and 50 µM β-ME) and seeded in 24 well plates at a density of 3.5-5 Mio cells/well at 37°C and 7.5% CO_2. On day one, peptides at a concentration of 1 µg/ml (HLA class I) or 5 µg/ml (HLA class II) per peptide were added. For expansion of T cells against the 20mer mutated sequences, peptides were added at 10 µg/ml and the culture supplemented with 20 µg/mL Hiltonol (Hiltonol®, Oncovir, Washington, DC, USA) [16]. On day three, five, seven and nine, rhIL-2 (R&D) was added at a final concentration of 2 ng/ml. Additionally, on day

five, cells were split 1:3 and if necessary, again 1:2 at day nine. After twelve days, cells were collected and cell viability and number checked with an automated cell counter (Nucleo Counter NC-250) using an AO-DAPI staining reagent.

IFNγ ELISpot assay

IFNγ secretion by PBMCs in response to peptide re-stimulation was determined using ELISpot assay. For this, *in vitro* expanded cells were seeded at a density of 2×10^5 cells/well in ELIspot filter plates (MSHAN4B, Millipore) that were pre-coated overnight at 4°C with 5 µg/ml anti-IFNγ monoclonal antibody (clone 1-D1K mAb, MabTech). Prior to cell seeding, ELISpot plates were washed twice with 150 µl serum free media (SFM – IMDM supplemented with 1% PenStrep and 50 µM β-ME) and incubated for at least 1 hour at 37°C with 50 µl TCM for blocking unspecific binding.

Cells were stimulated in TCM in at least 2 replicates with individual HLA class I peptides at 1 µg/ml, HLA class II peptides at 5 µg/ml (**Suppl. Table S1B**) or mutated peptides at 50 µg/ml (**Suppl. Table S1C**), as described [10,16]. As a positive control, half of the cells were seeded and stimulated with 10 µg/ml Phytohemagglutinin-L (PHA-L). DMSO, the solvent used for peptide dilution, was used as the negative control at a concentration matching that of the peptides. Following stimulation for 26 hours at 37°C and 7.5% CO_2, cells were removed, plates were washed and the detection of IFNγ was performed by the addition of the biotinylated anti-human IFNγ mAb (clone 7-B6-1 MabTech) for two hours, incubation with ExtrAvidin Alkaline Phosphatase for one hour and addition of the substrate BCIP/NBT to the wells for 4-7 minutes at room temperature (RT). All washing steps were performed as described [10,13]. Spots were scanned and counted with an ImmunoSpot Series 6 Core ELISpot Reader (C.T.L.). T cell responses were defined as follows: average of spots in the peptide condition of at least 7/100.000 seeded cells and at least two-fold above the average of spots in the negative control. Moreover, a statistical test was performed whenever three replicates were available [18].

Intracellular cytokine staining (ICS)

Cells collected after 12 day-IVS (PBMCs) or after culture of tumor fragments (TILs) were seeded at a density of $0.5\text{-}2\times10^6$ cells/well in a round bottom 96-well plate. For TILs, TNF and IFNγ production was assessed, while testing of PBMC reactivity to the peptides contained in the 2nd vaccine as well as to the mutated 20mer peptides additionally included the expression of CD107a, CD154 and IL-2 (for details on the mAb panels, see **Suppl. Table S2**). Briefly, post IVS cells were stimulated with the individual peptides (10 µg/ml for HLA class I and HLA class II, 50 µg/ml for 20mer mutated sequences) for one hour in TCM at 37°C and 7.5% CO_2

in the presence of 1.5 µl of CD107a-FITC mAb. After incubation, 10 µg/ml brefeldin A (Sigma-Aldrich) and monensin (GolgiStop, BD Biosciences) at a dilution of 1:1500 were added and cells were further incubated for 14 hours. In the case of the TILs, where no CD107a expression was investigated, peptides were added simultaneously to the brefeldin A and monensin and cells incubated for 12 hours. Water/DMSO and Staphylococcus enterotoxin B (SEB, 10 µg/ml) or phorbol myristate acetate + ionomycin (PMA 10 µg/ml /Iono 1 µM, both from Sigma) were used as negative and positive controls, respectively. After stimulation, cells were washed and stained for 20 minutes at 4°C with all mAbs against extracellular markers (**Suppl. Table S2**) together with a live cell dye diluted in FACS buffer (PBS supplemented with 2% hi fetal bovine serum (FBS), 0.02% NaN_3 and 2 mM EDTA). After one washing step with FACS buffer, cells were fixed and permeabilized with Cytofix/Cytoperm (BD Biosciences) for 20 minutes at 4°C followed by one washing step and staining with mAbs against the intracellular markers (**Suppl. Table S2**) diluted in Perm wash buffer (PBS supplemented with 0.02% NaN_3, 0.5% bovine serum albumin (BSA) and 0.1% saponin) for 20 minutes at 4°C. After two washes, samples were analyzed on a LSR Fortessa™ SORP Cell Analyzer (BD). At least $4x10^5$ cells were acquired. Data was analyzed using FlowJow v10. Results are presented as percentage of $marker^+$ cells within the $CD4^+$ or $CD8^+$ cells for HLA class II and HLA class I peptide stimulations, respectively, or in both for mutated peptides.

Immunohistochemistry (IHC)

Tissue sections with 2,5 µm thickness were shaved from paraffin embedded diagnostic tumor material obtained after surgical resections (L06/10; L03/11; L04/12; P03/13; L06/16). Hematoxylin-eosin (H&E) staining was performed using a standardized protocol. Immunohistochemical staining was performed on an automated immunostainer, following the manufacturer's protocol (Benchmark ULTRA, Ventana Medical Systems, Oro Valley, AZ) with DISCOVERY CC1 cell conditioning solution (Roche, Grenzach-Wyhlen, Germany) and optiView DAB IHC Detection Kit (Roche). Respective commercially available antibodies used, sources and dilutions used are provided in **Suppl. Table S3**. Sections were assessed by pathologists and annotated respectively.

Supplementary Tables

Table S1: Synthetic peptides

Table S1A: Vaccine peptides (1st vaccine - V)					
	**Peptide Sequence**	**Gene ID**	**AA-positions**	**HLA**	**Reference**
1	GLASFKSFLK	RGS-5	74-83	**A*03**	[10]
2	SLLTSSKGQLQK	ADFP (PLIN2)	369-380	**A*03**	[10]
3	TSALPIIQK	ADFP (PLIN2)	63-71	**A*03**	[10]
4	SLFPNSPKWTSK	MMP7	79-90	**A*03**	[10]
5	NPPSMVAAGSVVAAV	CCND1	198-212	**DR11** / DR4	[10,19,20]
6	HSKIIIIKKGHAKDSQ	IGFBP3	142-157	**DR11**	[10,19]
7	SQDDIKGIQKLYGKRS	MMP7	153-168	DR12 / **DQ7**	[10,19]

Table S1B: Vaccine peptides (2nd vaccine – V_{XS15})					
	**Peptide Sequence•**	**Gene ID**	**AA-positions**	**HLA**	**Reference**
1	**GLASFKSFLK**	**RGS-5**	74-83	**A*03**	[10]
2	**SLFPNSPKWTSK**	**MMP7**	79-90	**A*03**	[10]
3	FVQENYLEY	MAGEA3/MAGEA6	250-258	**A*29 / B*35**	n.a.
4	SPIDPSEKY	CATSPERG	84-92	**B*35**	n.a.
5	TPRVTGGGAM	CMV pp65	417-26	**B*07**	[11]
6	**NPPSMVAAGSVVAAV**	**CCND1**	198-212	**DR11** / DR4	[10,19,20]
7	**HSKIIIIKKGHAKDSQ**	**IGFBP3**	142-157	**DR11**	[10,19]
8	TLGEFLKLDRERAKN	BIRC5	97-11	**DR1**	[13]

•Peptides also included in the 1st vaccine are shown in bold; further the HLA-typing of the patient congruent with respective peptide presentation is marked in bold. n.a. stands for non-applicable, since peptides have not been previously published.

Table S1C: Gene variant-derived peptides			
	**Peptide Sequence***	**Gene ID**	**AA-positions**
1	NIPRLVSGWVKPIIIG**C**HAY	IDH1	116-135 (R132C)
2	VVNNIPFLRNAIMSY**L**LTSR	PTGS2	87-106 (V102L)
3	PNVPFPPTSNGLSGYK**G**SSH	KMT2C	4166-4185 (D4182G)
4	CINAGNTHYCQP**R**GYTGSY	NOTCH2	1126-1145 (L1139R)
5	YRGNAFPVEI**P**RRDDLVERP	POLE	248-267 (T258P)

** AA-exchanges predicted from DNA mutations are shown in bold.*

Table S2: mAbs panels for ICS

Table S2A: ICS – TILs -					
**#**	**Ab (clone) and dead cell dye**	**Fluorochrome**	**Company***	**Dilution**	**Staining step**
1	CD4 (RPA-T4)	APC-Cy7	BD	1:200	extracellular
2	CD8 (SFCI21Thy2D3)	PE-Cy7	BC	1:400	extracellular
3	Anti-IFNγ (B27)	FITC	BD	1:200	intracellular
4	Anti-TNF (Mab11)	Pacific Blue	BL	1:120	intracellular
5	Aqua Live Dead	-	IV	1:100	extracellular

Table S2B: ICS – PBMCs -					
**#**	**Ab (clone) and dead cell dye**	**Fluorochrome**	**Company***	**Dilution**	**Staining step**
1	CD107a (H4A3)	FITC	BD	1:100	culture
2	CD14 (63D3)	PerCP	BL	1:20	extracellular
3	CD19 (HIB19)	PerCP	BL	1:40	extracellular
4	CD4 (RPA-T4)	APC-Cy7	BD	1:100	extracellular
5	CD8 (RPA-T8)	BV605	BD	1:80	extracellular
6	Anti-IFNγ (B27)	Alexa Fluor 700	BL	1:200	intracellular
7	Anti-TNF (Mab11)	Pacific Blue	BL	1:200	intracellular
8	Anti-IL-2 (MQ1-17H12)	PE-Cy7	BL	1:20	intracellular
9	CD154 (24-31)	APC	BL	1:170	intracellular
10	Zombie Aqua	-	BL	1:200	extracellular

** BD = Becton Dickinson Biosciences. BC = Beckman Coulter. BL = Biolegend. IV = Invitrogen.*

Table S3: Abs used for IHC

Antibody	Clone	Supplier	Dilution
CD3	2GV6	Roche, Grenzach-Wyhlen, Germany	ready to use
CD4	SP35	Zytomed, Berlin, Germany	1:50
CD8	C8/114B	Agilent, Santa Clara, CA	1:300
CD68	KP1	Agilent, Santa Clara, CA	1:5000
CD163	MRQ-26	Cell Marque, Merck / MiliporeSigma, Burlington, MA	1:150
FOXP3	236A/E7	Abcam, Cambridge, UK	1:50
HLA-DR	TAL.1B5	Agilent, Santa Clara, CA	1:200
PD-L1	22C3	DAKO, Glostrup, Denmark	1:50

Supplementary Figures

Suppl. Figure S1

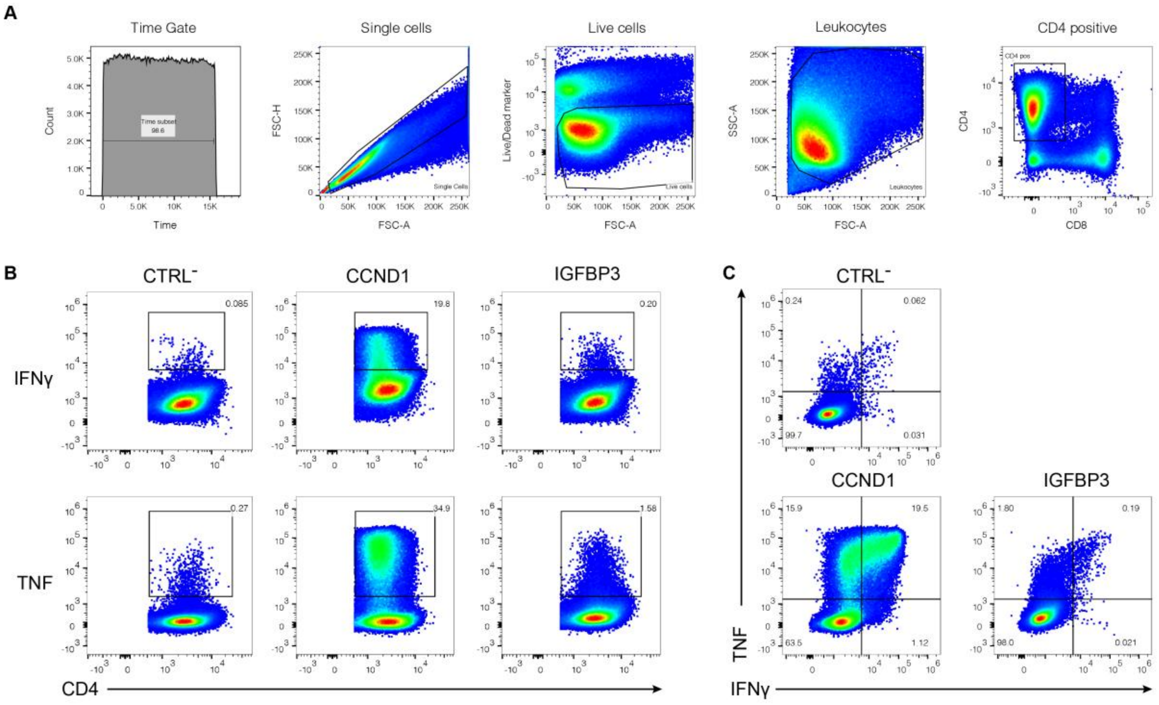

Supplementary Figure S1. **Vaccine (V)-specific T cells in TILs.** Gating strategy for defining cytokine-producing cells. **A**: Time, single cells, live cells, leukocytes and CD4 positive cell gates were applied. **B**: Within the $CD4^+$ cells, the % of cytokine-producing cells (top panel IFNγ, lower panel TNF) was determined. **C**: Representative dot-plots showing the $CD4^+$ T cells that produce TNF, IFNγ or both cytokines.

Suppl. Figure S2

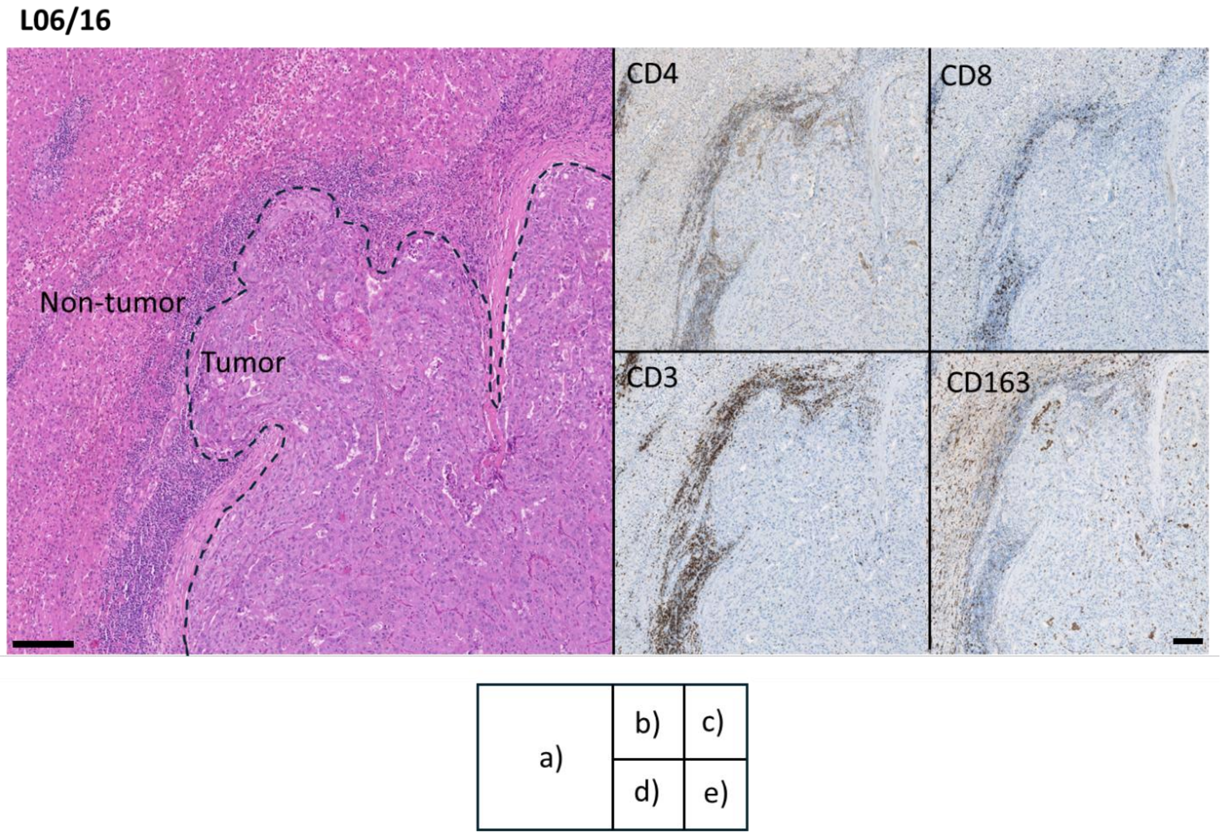

Supplementary Figure S2. H&E (left) and IHC (right) staining of recurrent liver tumor (L06/16) with annotated tumor invasive front (dashed line) in H&E staining (**a**) as well as respective IHC staining for CD4 (**b**), CD8 (**c**), CD3 (**d**) and CD163 (**e**) for a macrophage/ monocyte marker; (scale bar: 200µm).

Suppl. Figure S3

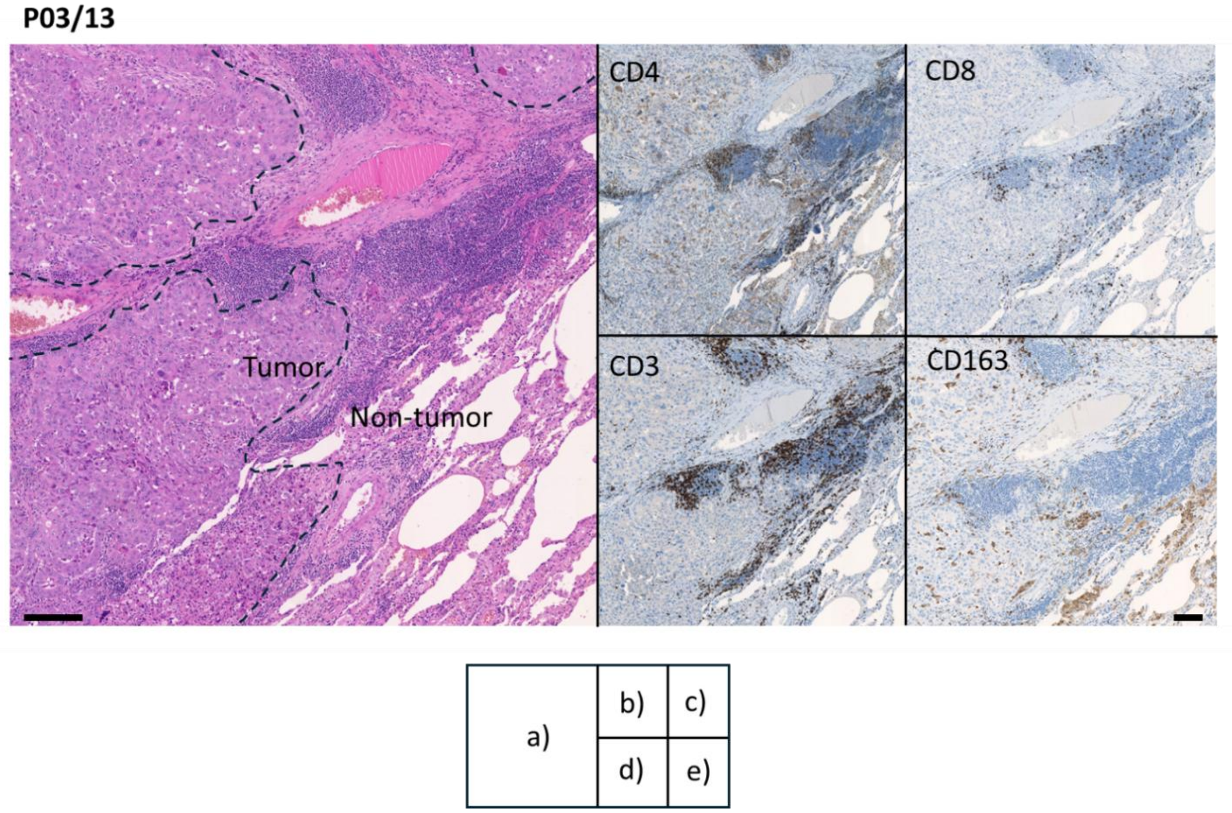

Supplementary Figure S3. H&E (left) and IHC (right) staining of pulmonary metastasis (P03/13) with annotated tumor invasive front (dashed line) in H&E staining (**a**) as well as respective IHC staining for CD4 (**b**), CD8 (**c**), CD3 (**d**) and CD163 (**e**) for a macrophage/ monocyte marker; (scale bar: 200µm).

Suppl. Figure S4

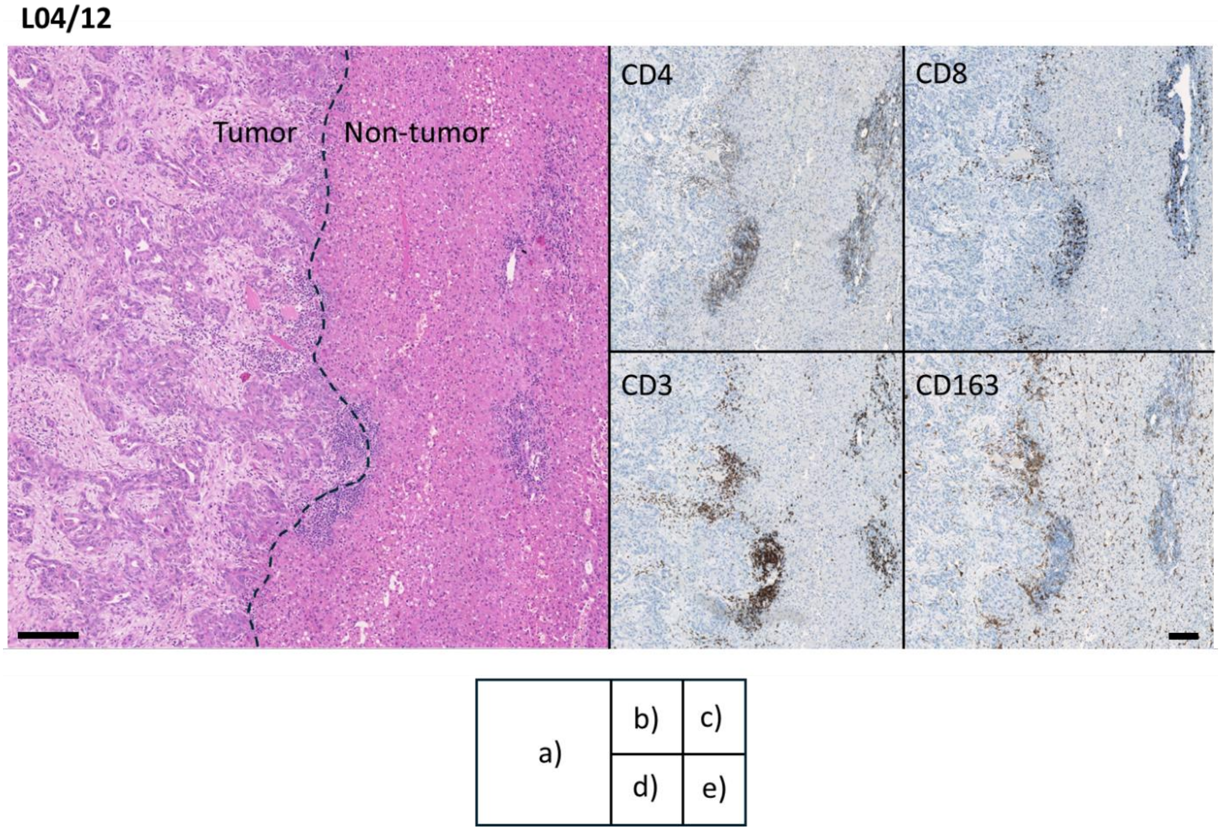

Supplementary Figure S4. H&E (left) and IHC (right) staining of recurrent liver tumor (L04/12) with annotated tumor invasive front (dashed line) in H&E staining (**a**) as well as respective IHC staining for CD4 (**b**), CD8 (**c**), CD3 (**d**) and CD163 (**e**) for a macrophage/ monocyte marker; (scale bar: 200µm).

Suppl. Figure S5

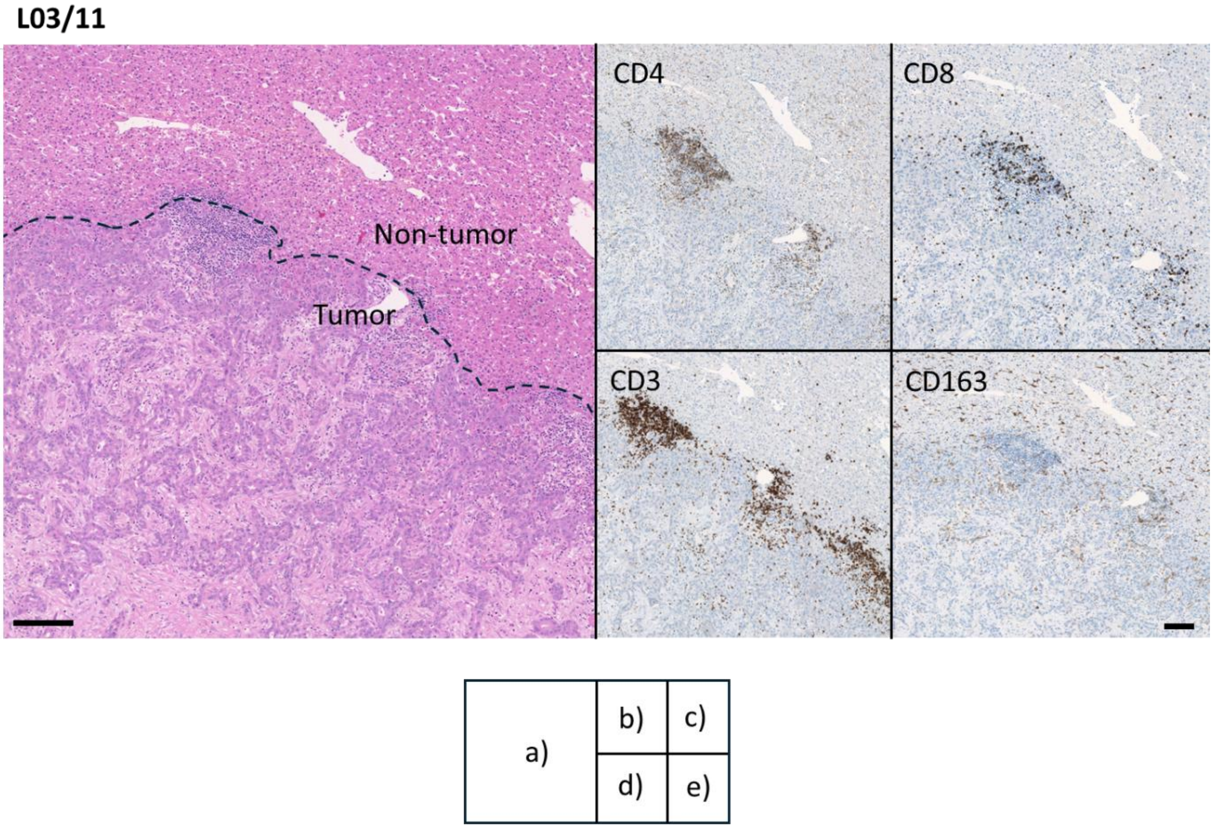

Supplementary Figure S5. H&E (left) and IHC (right) staining of recurrent liver tumor (L03/11) with annotated tumor invasive front (dashed line) in H&E staining (**a**) as well as respective IHC staining for CD4 (**b**), CD8 (**c**), CD3 (**d**) and CD163 (**e**) for a macrophage/ monocyte marker; (scale bar: 200µm).

Suppl. Figure S6

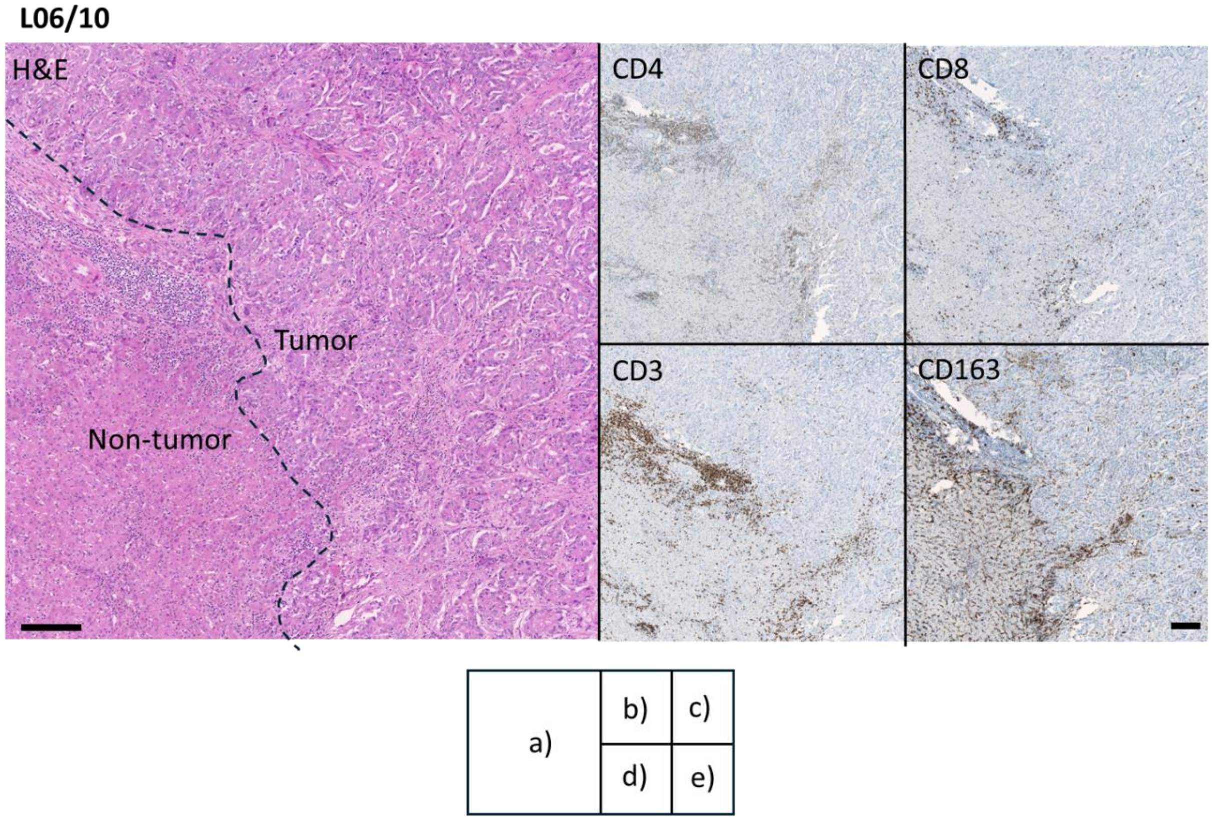

Supplementary Figure S6. H&E (left) and IHC (right) staining of primary liver tumor (L06/10) with annotated tumor invasive front (dashed line) in H&E staining (**a**) as well as respective IHC staining for CD4 (**b**), CD8 (**c**), CD3 (**d**) and CD163 (**e**) for a macrophage/ monocyte marker; (scale bar: 200µm).

Suppl. Figure S7

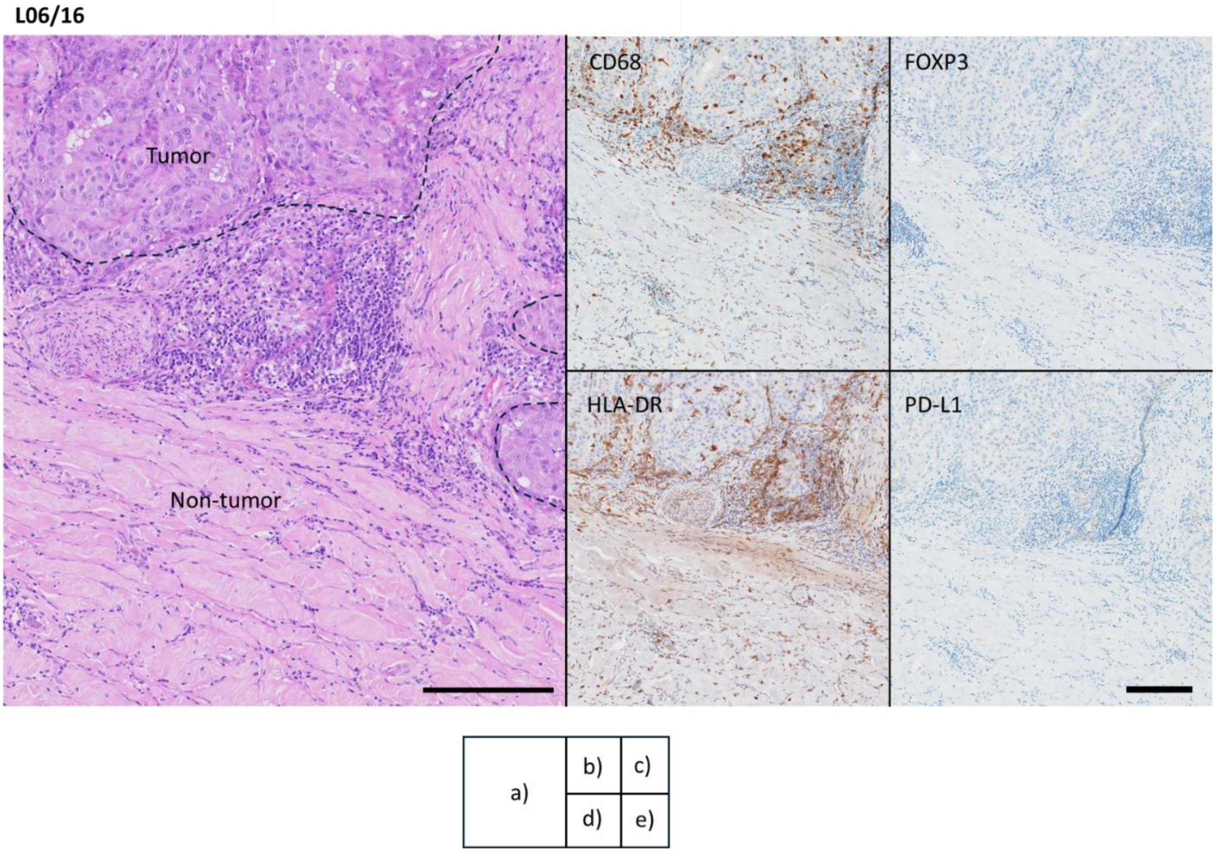

Supplementary Figure S7. H&E (left) and IHC (right) staining of recurrent liver tumor (L06/16) with annotated tumor invasive front (dashed line) in H&E staining (**a**) as well as respective IHC staining for CD68 for a macrophage marker (**b**), FOXP3 found in regulatory T cells (**c**), HLA-DR (**d**) and the immune checkpoint protein PD-L1 (**e**); (scale bar: 200µm).

Suppl. Figure S8

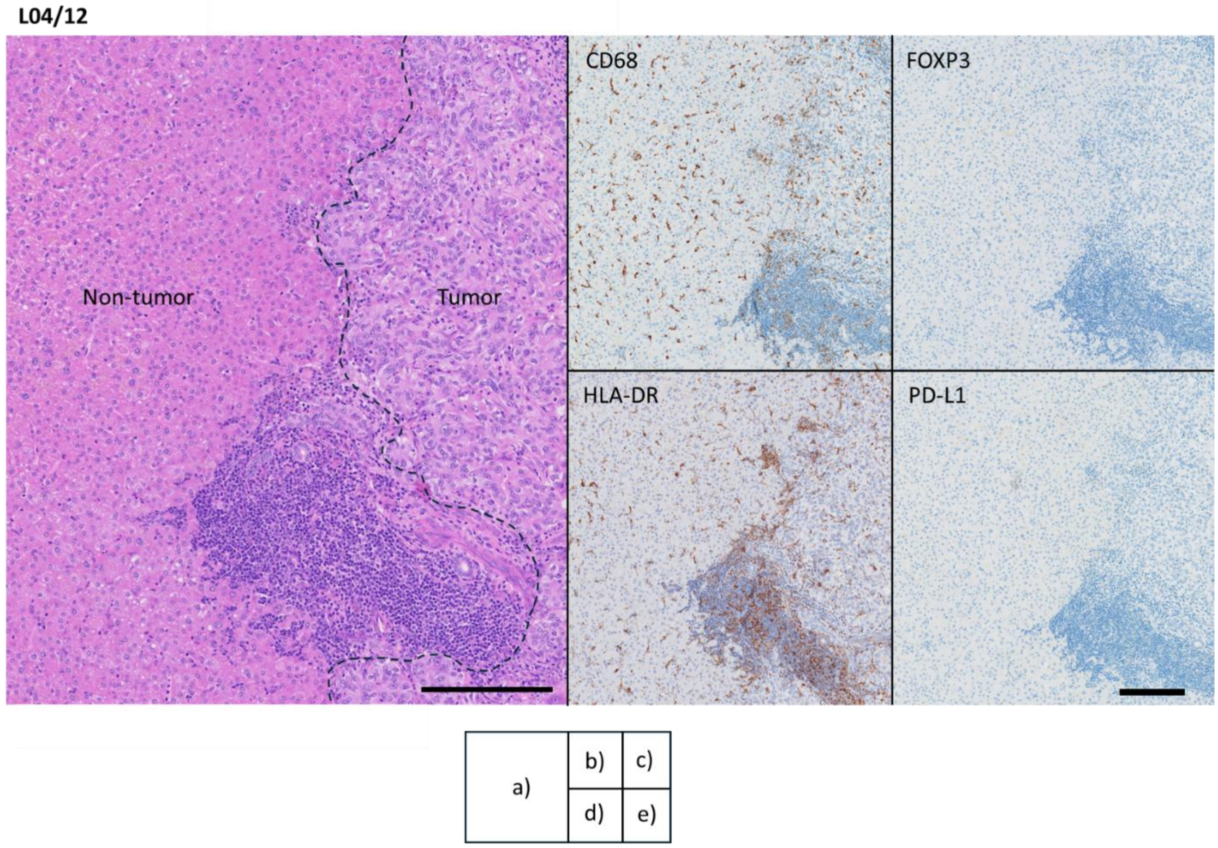

Supplementary Figure S8. H&E (left) and IHC (right) staining of recurrent liver tumor (L04/12) with annotated tumor invasive front (dashed line) in H&E staining (**a**) as well as respective IHC staining for CD68 for a macrophage marker (**b**), FOXP3 found in regulatory T cells (**c**), HLA-DR (**d**) and the immune checkpoint protein PD-L1 (**e**); (scale bar: 200µm).

Suppl. Figure S9

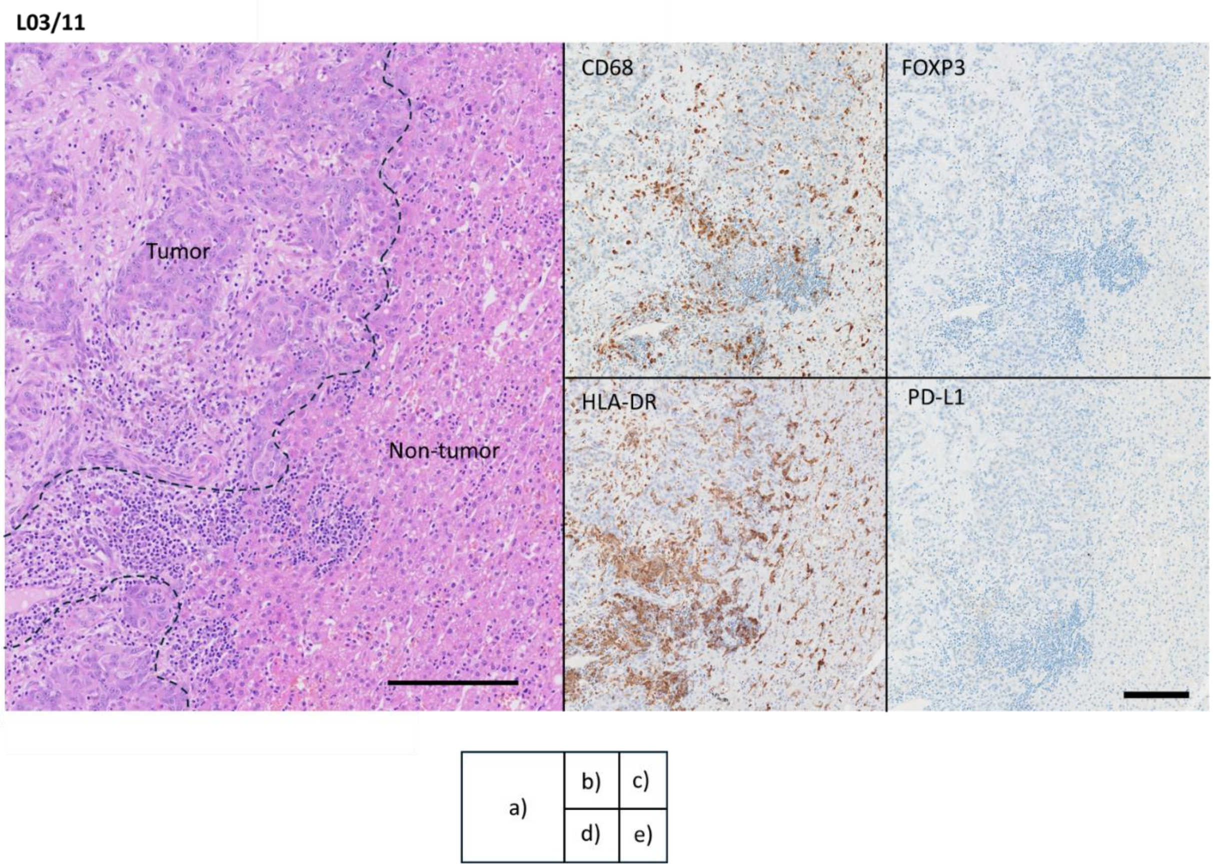

Supplementary Figure S9. **H&E (left) and IHC (right) staining of recurrent liver tumor (L03/11)** with annotated tumor invasive front (dashed line) in H&E staining (**a**) as well as respective IHC staining for CD68 for a macrophage marker (**b**), FOXP3 found in regulatory T cells (**c**), HLA-DR (**d**) and the immune checkpoint protein PD-L1 (**e**); (scale bar: 200µm).

Suppl. Figure S10

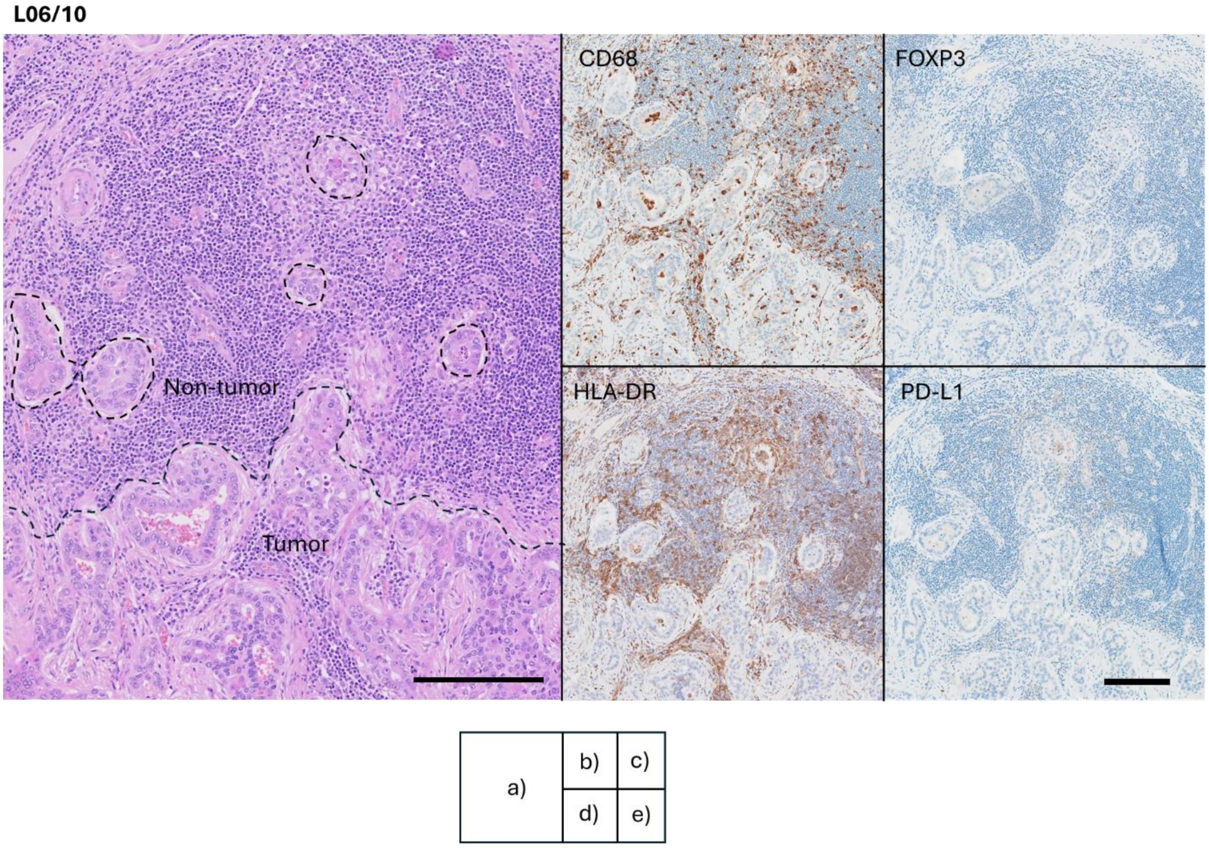

Supplementary Figure S10. H&E (left) and IHC (right) staining of primary liver tumor (L06/10) with annotated tumor invasive front (dashed line) in H&E staining (**a**) as well as respective IHC staining for CD68 for a macrophage marker (**b**), FOXP3 found in regulatory T cells (**c**), HLA-DR (**d**) and the immune checkpoint protein PD-L1 (**e**); (scale bar: 200µm).

Suppl. Figure S11

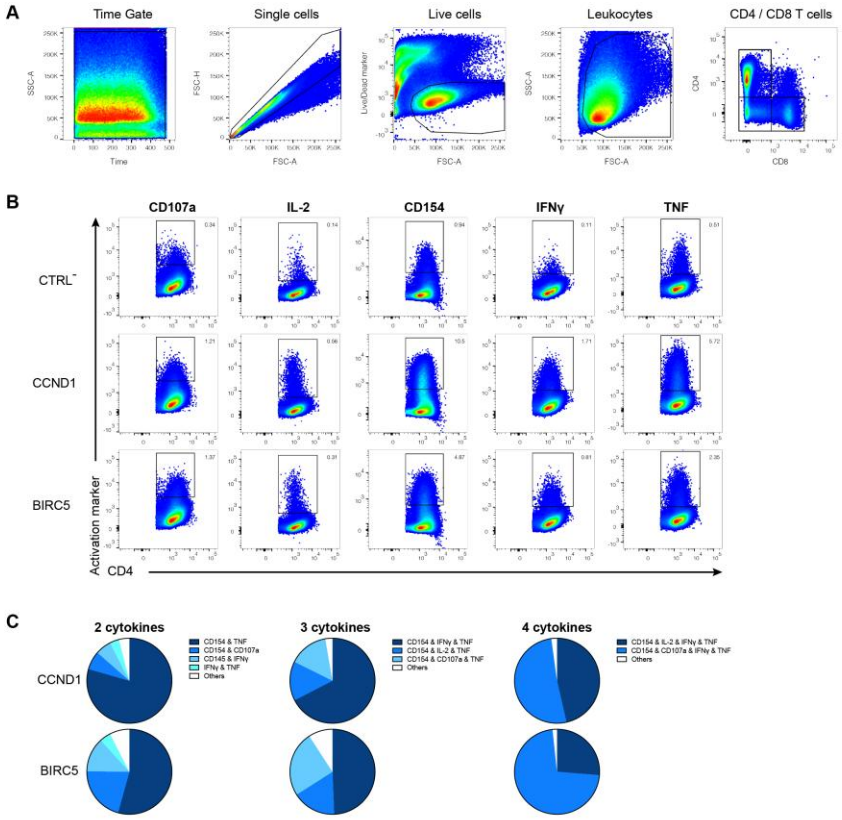

Supplementary Figure S11. T cell responses to HLA class II peptides after application of the 2nd vaccine. **A**: Gating strategy for defining activation marker-producing cells. Time, single cells, live cells, leukocytes and CD4 positive cell gates were applied. **B**: Within the $CD4^+$ cells, the % of activation marker-producing cells (CD107a, IL-2, CD154, IFNγ and TNF) is shown for CCND1 and BIRC5. **C**: Analyses of the most common combinations of expressed activation markers when the cells (V_{XS15} 4, after 4 vaccinations) produce 2, 3 or 4 activation markers after re-exposure to the peptide (CCND1 or BIRC5 shown).

Suppl. Figure S12

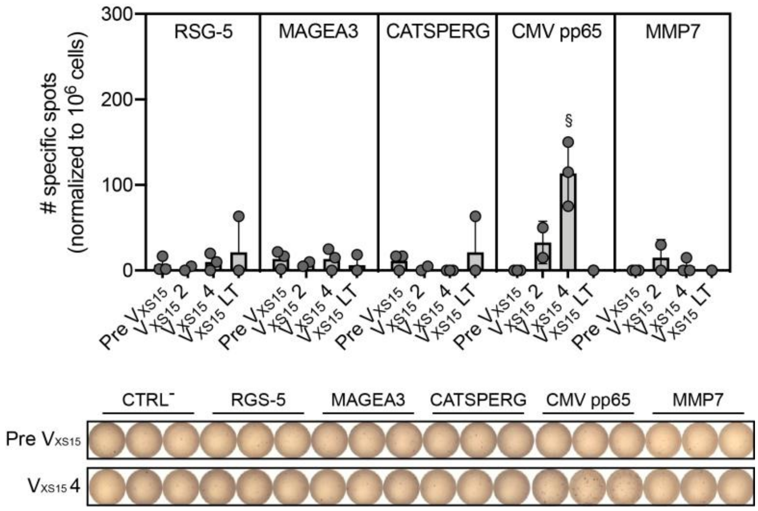

Supplementary Figure S12. T cell response to HLA class I peptides of the 2nd vaccine. Quantification and representative wells of the IFNγ ELISpot with short peptides (RGS-5, MAGEA3, CATSPERG, CMV pp65, MMP7). PBMCs were stimulated *in vitro* with the peptides for 12 days prior to IFNγ ELISpot analyses. Graph shows specific spots per 10^6 cells (background (CTRL$^-$) subtracted) in a time point prior to the vaccination (Pre V_{XS15}), after 2 (V_{XS15} 2) and 4 (V_{XS15} 4) vaccinations and in the long-term (V_{XS15} LT, 5 years after last vaccination). § marks conditions that were considered positive (see Materials and Methods for positivity criteria). Each condition was investigated in triplicates, except for V_{XS15} 2 where duplicates were done (each well is shown as a dot in the graph and bars represent means).

Suppl. Figure S13

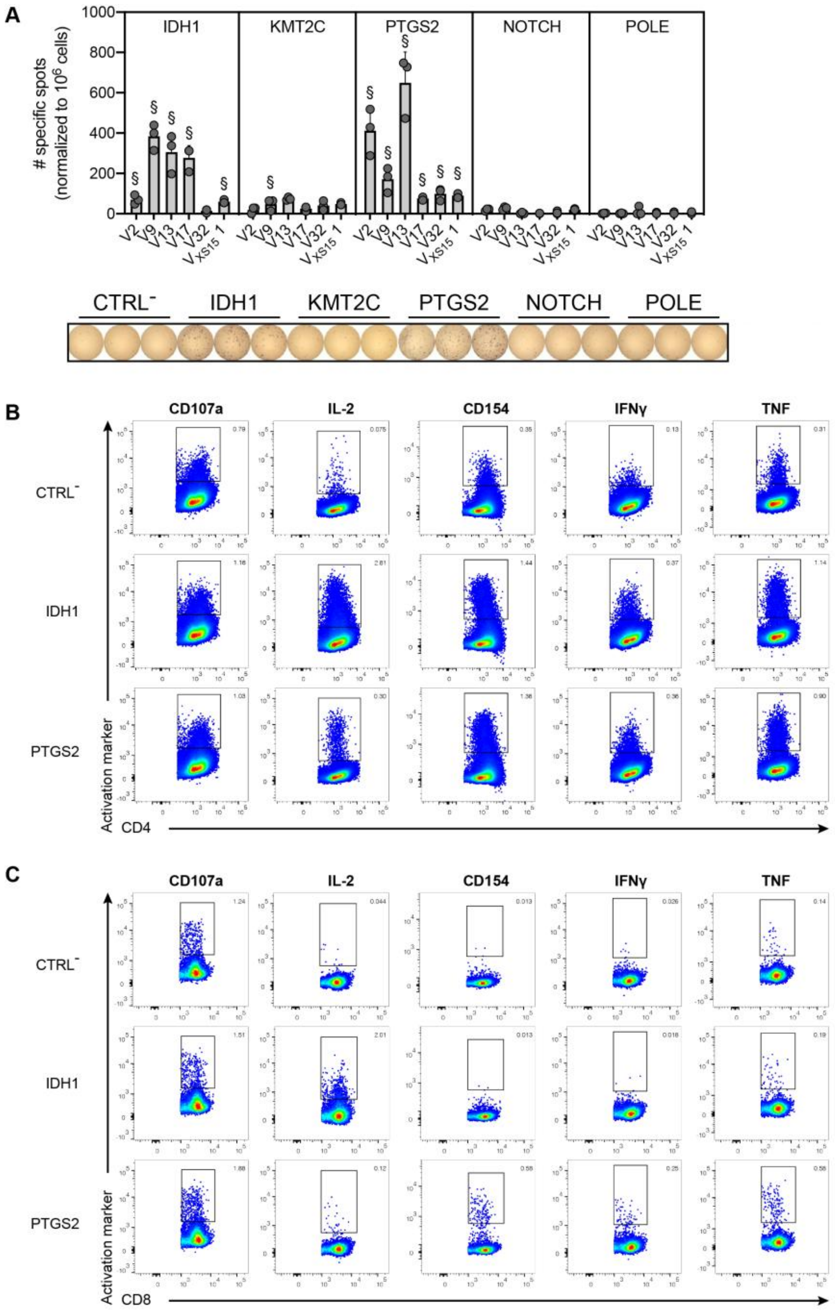

Supplementary Figure S13. T cell responses to mutated peptides. **A**: IFNγ ELISpot with 20mer peptides covering the different mutations that were identified in the patient´s tumor samples (IDH1$_{R132C}$, KMT2C$_{D4182G}$, PTGS2$_{V102L}$, NOTCH$_{L1139R}$, POLE$_{T258P}$, see **Suppl. Table S1C**). Graph shows specific spots per 10^6 cells (background (CTRL$^-$) subtracted) for the different time points (4.5 years in between). § marks conditions that were considered positive (see Materials and Methods for positivity criteria). Each condition was investigated in triplicates (each well is shown as a dot in the graph). Representative wells from timepoint V9 are shown. **B** and **C**: Representative dot-plots of activation marker-producing cells (CD107a, IL-2, CD154, IFNγ and TNF) in CD4$^+$ (**B**) or CD8$^+$ (**C**) T cells after re-exposure to peptides IDH1$_{R132C}$ or PTGS2$_{V102L}$.

References

1. Sturm M, Schroeder C, Bauer P. SeqPurge: highly-sensitive adapter trimming for paired-end NGS data. BMC Bioinform. 2016;17:208.

2. Faust GG, Hall IM. SAMBLASTER: fast duplicate marking and structural variant read extraction. Bioinformatics. 2014;30:2503–5.

3. Chen X, Schulz-Trieglaff O, Shaw R, Barnes B, Schlesinger F, Källberg M, et al. Manta: rapid detection of structural variants and indels for germline and cancer sequencing applications. Bioinformatics. 2015;32:1220–2.

4. Kim S, Scheffler K, Halpern AL, Bekritsky MA, Noh E, Källberg M, et al. Strelka2: fast and accurate calling of germline and somatic variants. Nat Methods. 2018;15:591–4.

5. Cingolani P, Platts A, Wang LL, Coon M, Nguyen T, Wang L, et al. A program for annotating and predicting the effects of single nucleotide polymorphisms, SnpEff. Fly. 2012;6:80–92.

6. Nelde A, Kowalewski DJ, Stevanović S. Antigen Processing, Methods and Protocols. Methods Mol Biol. 2019;1988:123–36.

7. Eng JK, McCormack AL, Yates JR. An approach to correlate tandem mass spectral data of peptides with amino acid sequences in a protein database. J Am Soc Mass Spectrom. 1994;5:976–89.

8. Käll L, Canterbury JD, Weston J, Noble WS, MacCoss MJ. Semi-supervised learning for peptide identification from shotgun proteomics datasets. Nat Methods. 2007;4:923–5.

9. Perez-Riverol Y, Csordas A, Bai J, Bernal-Llinares M, Hewapathirana S, Kundu DJ, et al. The PRIDE database and related tools and resources in 2019: improving support for quantification data. Nucleic Acids Res. 2019;47:D442–50.

10. Löffler MW, Chandran PA, Laske K, Schroeder C, Bonzheim I, Walzer M, et al. Personalized peptide vaccine-induced immune response associated with long-term survival of a metastatic cholangiocarcinoma patient. J Hepatol. 2016;65:849–55.

11. Weekes MP, Wills MR, Mynard K, Carmichael AJ, Sissons JGP. The Memory Cytotoxic T-Lymphocyte (CTL) Response to Human Cytomegalovirus Infection Contains Individual Peptide-Specific CTL Clones That Have Undergone Extensive Expansion In Vivo. J Virol. 1999;73:2099–108.

12. Löffler MW, Gori S, Izzo F, Mayer-Mokler A, Ascierto PA, Königsrainer A, et al. Phase I/II Multicenter Trial of a Novel Therapeutic Cancer Vaccine, HepaVac-101, for Hepatocellular Carcinoma. Clin Cancer Res. 2022;28:2555–66.

13. Widenmeyer M, Griesemann H, Stevanović S, Feyerabend S, Klein R, Attig S, et al. Promiscuous survivin peptide induces robust CD4+ T-cell responses in the majority of vaccinated cancer patients. Int J Cancer. 2012;131:140–9.

14. Rammensee H-G, Wiesmüller K-H, Chandran PA, Zelba H, Rusch E, Gouttefangeas C, et al. A new synthetic toll-like receptor 1/2 ligand is an efficient adjuvant for peptide vaccination in a human volunteer. J Immunother Cancer. 2019;7:307.

15. Behrendt R, White P, Offer J. Advances in Fmoc solid-phase peptide synthesis. J Pept Sci. 2016;22:4–27.

16. Schuhmacher J, Kleemann L, Richardson JR, Rusch E, Rammensee H-G, Gouttefangeas C. Simultaneous Identification of Functional Antigen-Specific CD8+ and CD4+ Cells after In Vitro Expansion Using Elongated Peptides. Cells. 2022;11:3451.

17. Löffler MW, Nussbaum B, Jäger G, Jurmeister PS, Budczies J, Pereira PL, et al. A Non-interventional Clinical Trial Assessing Immune Responses After Radiofrequency Ablation of Liver Metastases From Colorectal Cancer. Front Immunol. 2019;10:2526.

18. Moodie Z, Price L, Gouttefangeas C, Mander A, Janetzki S, Löwer M, et al. Response definition criteria for ELISPOT assays revisited. Cancer Immunol, Immunother. 2010;59:1489–501.

19. Marcu A, Bichmann L, Kuchenbecker L, Kowalewski DJ, Freudenmann LK, Backert L, et al. HLA Ligand Atlas: a benign reference of HLA-presented peptides to improve T-cell-based cancer immunotherapy. J Immunother Cancer. 2021;9(4):e002071.

20. Dengjel J, Decker P, Schoor O, Altenberend F, Weinschenk T, Rammensee HG, et al. Identification of a naturally processed cyclin D1 T-helper epitope by a novel combination of HLA class II targeting and differential mass spectrometry. Eur J Immunol. 2004;34(12):3644-51.
